# Structural commonalities determined by physicochemical principles in the complex polymorphism of the amyloid state of proteins

**DOI:** 10.1042/BCJ20240602

**Published:** 2025-01-22

**Authors:** Silvia Errico, Giulia Fani, Salvador Ventura, Joost Schymkowitz, Frederic Rousseau, Antonio Trovato, Michele Vendruscolo, Francesco Bemporad, Fabrizio Chiti

**Affiliations:** 1 Department of Experimental and Clinical Biomedical Sciences “Mario Serio”, Section of Biochemistry, University of Florence, 50134 Florence, Italy; 2 Institut de Biotecnologia i de Biomedicina (IBB) and Departament de Bioquímica i Biologia Molecular, Universitat Autònoma de Barcelona, 08193 Bellaterra, Barcelona, Spain; 3 Switch Laboratory, VIB Center for Brain and Disease Research, 3000 Leuven, Belgium; 4 Switch Laboratory, Department of Cellular and Molecular Medicine, KU Leuven, 3000 Leuven, Belgium; 5 Switch Laboratory, VIB Center for AI & Computational Biology, 3000 Leuven, Belgium; 6 Department of Physics and Astronomy “G. Galilei”, University of Padova, 35131 Padova, Italy; 7 National Institute of Nuclear Physics (INFN), Padova Section, University of Padova, 35131 Padova, Italy; 8 Centre for Misfolding Diseases, Yusuf Hamied Department of Chemistry, University of Cambridge, CB21EW Cambridge, U.K.

**Keywords:** Aggregation score, Amyloid predictors, Aggregation hot spots, Amyloid hot spots, Aggregation propensity profile, Amyloid prediction

## Abstract

Advances in solid-state nuclear magnetic resonance (ssNMR) spectroscopy and cryogenic electron microscopy (cryoEM) have revealed the polymorphic nature of the amyloid state of proteins. Given the association of amyloid with protein misfolding disorders, it is important to understand the principles underlying this polymorphism. To address this problem, we combined computational tools to predict the specific regions of the sequence forming the β-spine of amyloid fibrils with the availability of 30, 83 and 24 amyloid structures deposited in the *Protein Data Bank* (PDB) and *Amyloid Atlas* (AAt) for the amyloid β (Aβ) peptide, α-synuclein (αS), and the 4R isoforms of tau, associated with Alzheimer’s disease, Parkinson’s disease, and various tauopathies, respectively. This approach enabled a statistical analysis of sequences forming β-sheet regions in amyloid polymorphs. We computed for any given sequence residue *n* the fraction of PDB/AAt structures in which that residue adopts a β-sheet conformation (*F*_β_(*n*)) to generate an experimental, structure-based profile of *F_β_(n) vs n*, which represents the β-conformational preference of any residue in the amyloid state. The peaks in the respective *F*_β_(*n*) profiles of the three proteins, corresponding to sequence regions adopting more frequently the β-sheet structural core in the various fibrillar structures, align very well with the peaks identified with five predictive algorithms (ZYGGREGATOR, TANGO, PASTA, AGGRESCAN, and WALTZ). These results indicate that, despite amyloid polymorphism, sequence regions most often forming the structural core of amyloid have high hydrophobicity, high intrinsic β-sheet propensity and low electrostatic charge across the sequence, as rationalised and predicted by the algorithms.

## Introduction

The ability of specific peptides and longer proteins, either globular or intrinsically disordered, to self-assemble into well-defined aggregates characterised by a fibrillar morphology, a cross-β structure and an ability to bind specific dyes, known as amyloid fibrils, has been known for over 50 years [1–4]. Until the end of the last century, it was thought that this ability was limited to around 20 proteins, identified as the main constituents of the amyloid deposits observed in well-defined pathological conditions, including Alzheimer’s disease (AD), Parkinson’s disease (PD), light chain amyloidosis (AL) and transthyretin amyloidosis (ATTR) [4,5]. In 1998, however, the SH3 domain from the p85α subunit of bovine phosphatidylinositol 3-kinase was serendipitously found to form, in a purified form and under appropriate conditions *in vitro*, amyloid fibrils that were morphologically, structurally and tinctorially indistinguishable from those associated with disease [6]. In the following years, many other peptides and proteins, now amounting to over 100, were converted into amyloid fibrils, leading to the concept that many proteins, if not all, have an intrinsic ability to form amyloid fibrils *in vitro* under appropriate conditions [7,8].

It soon became evident that all such proteins, whether associated with disease or not, did not use the entirety of their amino acid sequence to form the cross-β structural core of their corresponding amyloid fibrils, but rather short regions of the sequence [9–11]. For example, amino acid substitutions within the model protein human muscle acylphosphatase were able to change the aggregation rate only when occurring in two short regions of the sequence [9] and they did so based on the effects of the substitutions on the hydrophobicity, propensity to form β-sheet structure and charge of the sequence at the site of mutation [12]. This observation led to the definition of a scale of aggregation propensity values of the 20 natural amino acids found in proteins, based on their hydrophobicity, β-sheet propensity and charge and to the development of an algorithm, later named ZYGGREGATOR, that determines a residue-dependent aggregation propensity profile over an input sequence of interest averaging the values over 7-residue sliding windows. The algorithm was proposed to predict the regions of the sequence promoting amyloid fibril formation based on the peaks in the profile, as well as the effect of mutations [13–15].

In an independent study, another algorithm, named TANGO, was shown to identify the sequence regions promoting β-aggregation [16]. In this statistical mechanics algorithm, every sequence segment of a protein is scored on the grounds of its frequency to adopt a fully buried β-sheet conformation and its related energy, predicting β-aggregating segments by calculating the partition function of the conformational phase space [16]. Both TANGO and ZYGGREGATOR include the possibility to calculate the impact of extrinsic parameters, including pH, ionic strength, and protein concentration, on the aggregation propensity.

Another computational tool, PASTA 2.0, harnessed a database of globular proteins to compute the interaction energies for each pair of amino acids found to be hydrogen-bonded in β-sheets of folded functional proteins [17]. Assuming that amyloid fibrils originate from the stacking among identical stretches of different molecules, PASTA 2.0 uses the interaction energies to scan an input sequence and calculate the propensity of any stretch of the sequence to give rise to intermolecular β-sheets. Consequently, unlike other methods, PASTA 2.0 provides the opportunity to predict the parallel or antiparallel arrangement of the protein segments within the aggregate.

AGGRESCAN was the first algorithm to exploit exclusively experimental information collected in cells [18]. The authors generated a set of DNA sequence variants encoding amyloid β (Aβ), substituting Phe19 with all possible amino acids. The 20 peptide variants expressed in *Escherichia coli* as proteins fused to the green fluorescent protein (GFP-Aβ) were tested for their ability to aggregate intracellularly by measuring the associated GFP fluorescence. This resulted in a scale of aggregation propensity values of the 20 amino acids. The measured scores are thus assigned to the amino acids of any input sequence and the algorithm averages the profile over a 7-residue sliding window.

Another algorithm, WALTZ, was developed starting from a database of hexapeptides exhibiting different aggregation propensities, used to generate a position-specific scoring matrix [19]. This latter matrix, together with the physicochemical properties of β-amyloids and the conformational features of amyloid backbone structures, was used to build a scoring function. WALTZ is able to discriminate between ordered amyloid aggregates and amorphous β-sheet aggregates. Many other algorithms have concomitantly and since then been developed (reviewed in [20]). The five tools mentioned above were among the first that became soon widely used in academia and industry.

All these computational tools were tested on the few experimental data available at the time of their development, in particular on the sequence regions forming the cross-β core of the fibrils, including those identified by protein mutagenesis, limited proteolysis, hydrogen-deuterium exchange, electron paramagnetic resonance (EPR) spectroscopy and the first applications of solid-state nuclear magnetic resonance (NMR) spectroscopy. However, due to major technical advances of solid-state NMR (ssNMR) and, in particular, cryogenic electron microscopy (cryoEM), it was later possible to resolve amyloid fibril structure with unprecedented resolution, down to <3.0 Å with cryoEM, and identify with higher accuracy the boundaries of the β-strands in the fibrils. Alongside these advancements came the realisation that the same peptide or protein could lead to many different fibril structures, where different sequence segments would participate in the cross-β core [21,22]. This structural variability likely results from a lack of evolutionary pressure yielding a relatively flat free energy landscape, unlike functional amyloids that were evolutionary optimised and do not, therefore, present this structural heterogeneity, similarly to functional folded states [23]. The microenvironments in specific brain regions can selectively favour a limited set of disease-related amyloid structures, as well as different conditions of pH, temperature, solutes and other factors in studies *in vitro*. This restricted structural choice is often linked to distinct disease manifestations. Therefore, understanding how environmental factors influence amyloid assembly is crucial when designing diagnostic and therapeutic tools to combat diseases associated with protein aggregation.

This observation, referred to as fibril polymorphism, prompted the question of whether these computational tools could predict just one of the many structures that each polypeptide chain could adopt, or instead capture the polymorphic nature of the amyloid state. The polymorphism of the amyloid state of proteins is in stark contrast with the uniqueness of their folded state. Understanding which physicochemical principles underlie this difference is important both to understand the fundamental nature of the different states of proteins and in biomedical applications, as many latest therapeutic approaches for AD and PD target the amyloid state and therefore their efficacy may be affected by the specific polymorphic forms present in diseases [24–29].

To address this issue, we exploited the fact that the widespread use of ssNMR and cryoEM has significantly increased the number of amyloid structures available in the *Protein Data Bank* (PDB). This is especially true for the three most widely studied and perhaps paradigmatic proteins in the amyloid field, that are Aβ, α-synuclein (αS) and the 4R isoform of tau (4R tau), associated with AD, PD and tauopathies, respectively. In fact, as many as 32, 89 and 24 amyloid structures have been published in the PDB for the three proteins, respectively, all listed in the newly edited *Amyloid Atlas* [21], which can now be re-examined in a statistical manner. In this work, we quantified the frequency of individual residues and sequence regions of Aβ, αS and 4R tau that adopt a β-conformation across the diverse amyloid polymorphs documented in the *Amyloid Atlas*. This information has been used to edit an experimental, structure-based β-sheet preference profile by plotting the frequency of each residue (or segment) adopting a β-conformation in the various structures as a function of residue number. We show that even the earliest algorithms, published in 2004–2005, when none of these PDB structures had yet been reported, can predict the sequence segments of Aβ, αS and 4R tau where a β-conformation is statistically most represented for their residues in these amyloid fibril structures. These results identify the main physicochemical principles determining the wide distribution of polymorphs in the amyloid state of proteins.

## Results

### Different amyloid fibril polymorphs of Aβ have different β-strands

We started our analysis using the 30 structures of amyloid protofilaments or fibrils of the Aβ peptide that have been deposited in the PDB and collected in the *Amyloid Atlas* created by Eisenberg and coworkers [21]. To avoid any bias in the criteria of a possible pre-selection, we considered all 30 structures present in the *Amyloid Atlas*, corresponding to either Aβ_1-40_ or Aβ_1-42_, either wild-type or mutant forms (Supplementary Table S1). The *Amyloid Atlas* structures are actually 32, but we excluded the two structures obtained with synthetic fragments of Aβ spanning residues 20–34 (Aβ_20-34_) and corresponding to PDB entries 6oiz and 6nb9 because they report on an excessively small fraction of the Aβ sequence (<40%). 13 and 17 of the 30 structures correspond to Aβ_1-40_ and Aβ_1-42_, respectively (Supplementary Table S1). 22 and 8 were obtained with wild-type peptides or mutated/modified forms, respectively. 13 were determined using ssNMR, with the remainder obtained with cryoEM. The 30 structures in the dataset were determined by 19 different research groups (Supplementary Table S1).

**Figure 1:**
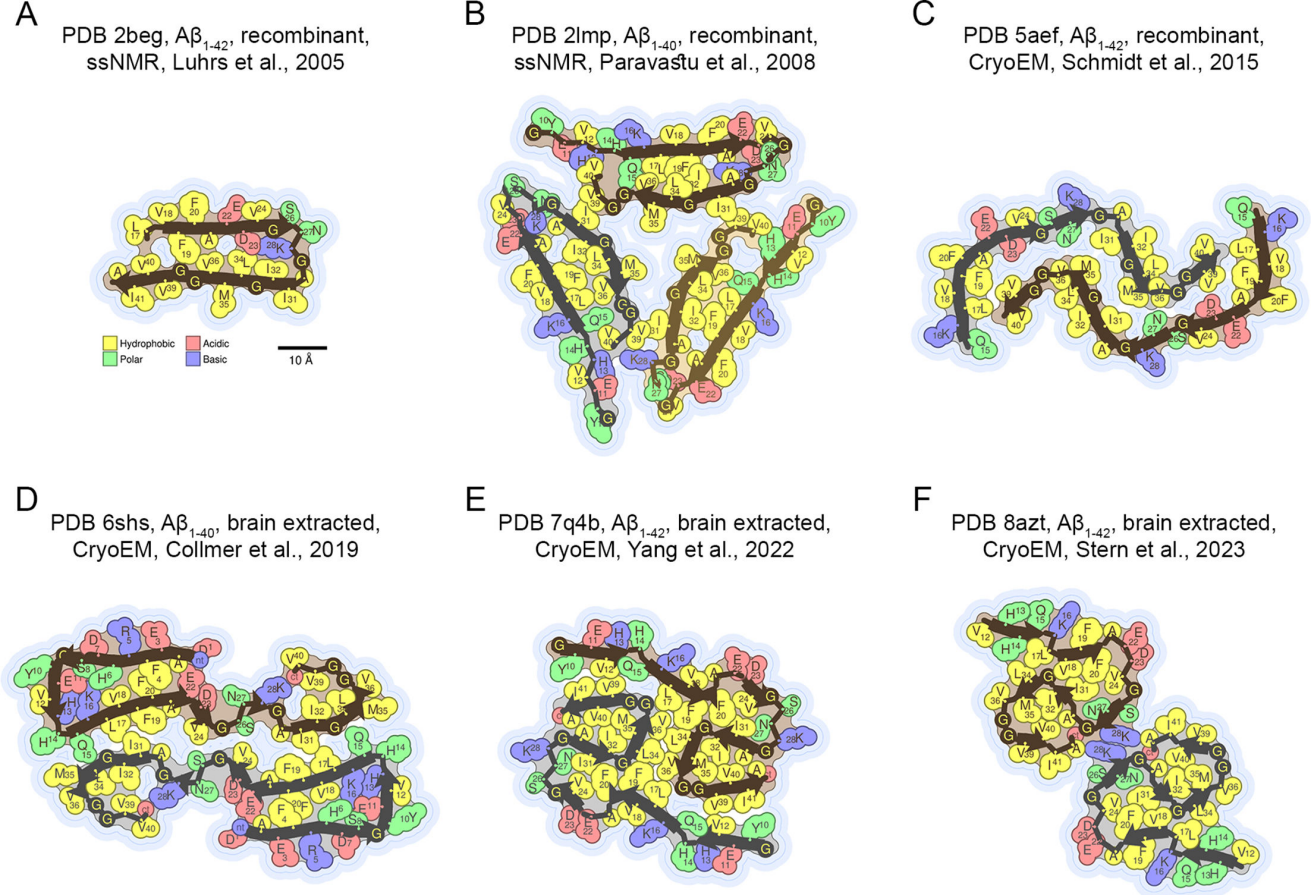
Representative amyloid fibril/protofilament structures of Aβ deposited in the PDB and listed in the *Amyloid Atlas* [21]. (**A-F**) The colour code and size bar reported in Panel A refer to all six Panels. In each case, the PDB entry, peptide length (Aβ_1-40_ or Aβ_1-42_), origin of analysed fibrils (recombinant or brain extracted), technique (ssNMR or cryoEM) and reference are reported. Each structure reports the residues adopting a β-sheet conformation (for example, residues 18–26 and 31–42 in the first structure of panel A).

Figure 1 reports six representative amyloid fibril structures out of this group of 30 analysed ones. The various structures were very diverse in terms of overall morphology, inter-residue contacts and residues involved in β-strand formation. In each case, the high resolution of the structure allowed to identify with good accuracy the amino acid residues adopting a β-sheet conformation based on the backbone dihedral angles ϕ and ψ assigned to the β-sheet secondary structure in the Ramachandran plot (see methods for more details). As an example, these correspond to residues 18–26 and 31–42 in the first structure illustrated in Figure 1A and corresponding to PDB entry 2beg.

By comparing the β-strand stretches in these ssNMR- or cryoEM-determined structures with the aggregation propensity profile of Aβ_1-42_ using the earliest version of ZYGGREGATOR [13], which was meant to identify the same stretches as residues 15–21 and 31–42 (*Z^prof^*_agg_ ≥ 1), we see that in some cases the agreement is good (Supplementary Figure S1A,B; *P* < 0.001, *κ* > 0.6), in some cases it is only partially satisfactory (SupplementaryFigure S1CE; *P* < 0.01, 0.4 < *κ* < 0.6) and in other cases it is poor (Supplementary Figure S1D,F; *P* > 0.05, *κ* < 0.4), as determined with the *P* value calculated with the one-tail Fisher’s exact test (FET) and *κ* value with the Cohen’s kappa test (CKT). This scenario does not change when using other algorithms, such as AGGRESCAN, TANGO, WALTZ and PASTA 2.0 (data not shown). The comparisons were extended to all 30 ssNMR- or cryoEM-determined structures of Aβ fibrils and to all five algorithms. The agreement between theoretical and experimental β-strands is reported for each case as *P* and *κ* values, and ranges from highly statistically significant to highly non-significant values with all five algorithms tested (Supplementary Tables S1 and S2). These discrepancies largely arise from the fibril polymorphism emerged experimentally with ssNMR and cryoEM, which involve different β-strands in the various cases. As a further analysis, box plots showing the distributions of κ values over all the available 30 structures and obtained with each of the five algorithms are shown in Supplementary Figure S7A (L*_β_* ≥ 4 AA) and Supplementary Figure S7B (L*_β_* ≥ 1 AA). They range for each of the five algorithms from low to high κ values, although in many cases and on average they are high, indicating that almost all algorithms have good agreement with one or more structures.

### Definition of a polymorph-independent, structure-based, β-sheet propensity profile for Aβ

In order to analyse all the 30 experimental structures together, for a given residue numbered *n* in the Aβ sequence, the fraction of structures out of all the 30 amyloid structures that contained the given residue in a β-sheet conformation was calculated (*F*_β_(*n*)). This value ranges from 0% to 100% if that residue adopts a β-conformation in none and all of the 30 structures, respectively. It will be 40%, for example, if it was found in a β-sheet conformation in 12 out of 30 structures (for residues 41 and 42, *F*_β_(*n*) was normalised to the 21 structures that contained those two residues). *F*_β_(*n*) is, therefore, a parameter that quantifies the β-conformational preference of any given residue *n* in the amyloid state, as it results from the close inspection of a statistically relevant number of amyloid structures.

**Figure 2:**
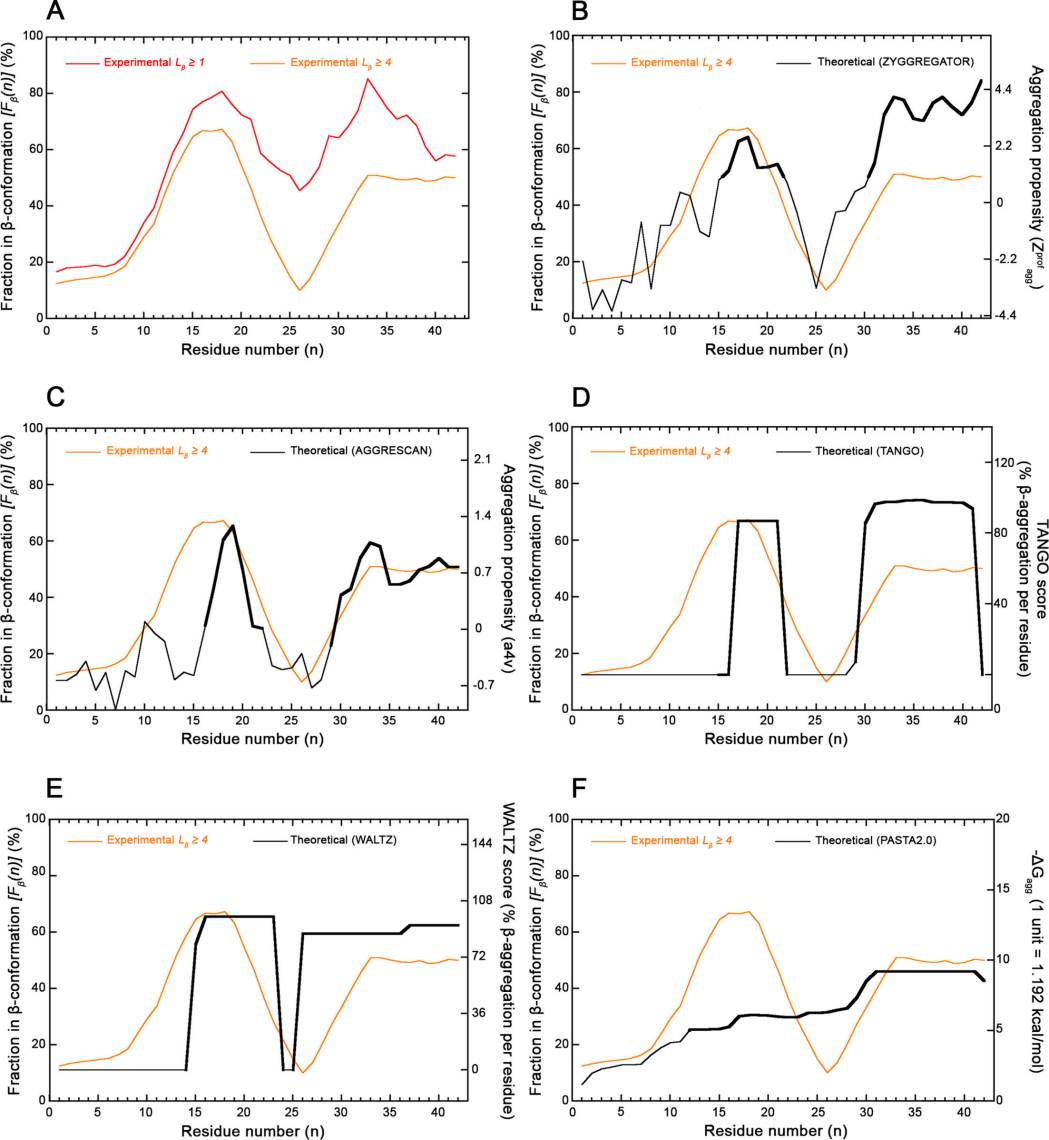
Experimental and theoretical profiles for the Aβ peptide. (**A**) Experimental, structure-based β-sheet propensity profile *(F*_*β*_*(n))* vs. residue number *n* determined with all 30 available amyloid fibril structures of Aβ_1-40_ or Aβ_1-42_ deposited in the PDB and listed in the *Amyloid Atlas* [21]. Profiles were edited by considering residues in the β-sheet conformation when belonging to all β-strands with *L*_β_ ≥ 1 AA (red) and *L*_β_ ≥ 4 AA (orange). (**B-F**) Structure-based *F*_β_(n) profile vs. residue number *n* obtained for *L*_β_ ≥ 4 AA (orange) compared with the predicted aggregation propensity profile (black) edited with ZYGGREGATOR (**B**), AGGRESCAN (**C**), TANGO (**D**), WALTZ (**E**) and PASTA (**F**) as described [13,16–19]. In all cases, parameters and units of the experimental structure-based (orange) and predicted algorithm-based (black) profiles are reported on the left and right *Y* axes, respectively. Sequence regions predicted theoretically to adopt a β-strand conformation (*Z^prof^_agg_* ≥ 1, *a4v* ≥ 0, *TANGO score* > 0, *WALTZ score* > 75%, *–∆G_agg_* > 5 units, respectively) are highlighted in bold.

A single, structure-based β-sheet propensity profile of the Aβ sequence in the amyloid state as a function of residue number *n* was generated by plotting *F*_β_(*n*), averaged over sliding windows of seven residues, versus *n* (Figure 2A, red line). The profile shows that the various residues over the sequence have different β-sheet preferences and that residues adopting more frequently a β-sheet conformation in the amyloid fibrils (*F*_β_(*n*) > 55%) are approximately residues 13–23 and 29–42 (Figure 2A, red line). When the analysis was repeated from the beginning by considering residues in a β-conformation only when present in β-strands with length *L_β_* ≥ 4 amino acid (AA) residues, the profile had, as expected, lower values of *F*_β_(*n*) over the sequence, but confirms that the various residues have different β-sheet preferences with the highest values (*F*_β_(*n*) ≥ 40%) around residues 12–21 and 31–42 (Figure 2A, orange line).

### Agreement between experimental structure-based and predicted sequence-based aggregation propensity profiles for Aβ

An extremely good agreement was found between the experimental, structure-based *F*_β_(*n*) profile and the predicted aggregation propensity profile obtained with the earliest version of the ZYGGREGATOR algorithm [13], as shown in Figure 2B and Supplementary Figure S2B. According to this previously published analysis, the algorithm predicted two regions of the sequence participating in the amyloid cross-β core of the fibrils, that is residues 15–21 and 31–42 (*Z^prof^*_agg_ ≥ 1), in extremely good agreement with the peaks 12–21 and 31–42 (*F*_β_(*n*) ≥ 40%) in the experimental *F*_β_(*n*) profile, obtained with *L_β_* ≥ 4 AA (Figure 2B, *P* < 0.001 with FET, *k* = 0.858 with CKT and Supplementary Figure S2B for *L_β_* ≥ 1 AA, *P* ≤ 0.001 with FET, *k* = 0.719 with CKT). Since the algorithm was published in 2005 and all of the 30 structures reported in the *Amyloid Atlas* were released over a later time window (years 2005–2023) and analysed *in toto* without any pre-selection or case exclusion, the agreement shows that the various residues of the Aβ peptide have a higher conformational preference towards a cross-β structure in the amyloid state when possessing a high hydrophobicity, high β-sheet propensity and low charge (Figure 2B and Supplementary Figure S2B). Such stretches appear, therefore, to be defined by their intrinsic physicochemical properties, being, thus, sequence-dependent rather than structure-dependent.

A very good agreement was also found between the experimental, structure-based *F*_β_(*n*) profile and the theoretical aggregation propensity profile obtained with AGGRESCAN [18], as shown in Figure 2C and Supplementary Figure S2C. AGGRESCAN predicted the two regions 17–22 and 29–42 (*a4v* ≥ *0*), in substantial agreement with the regions 12–21 and 31–42 obtained with *L_β_* ≥ 4 AA (*F*_β_(*n*) ≥ 40%) in the experimental *F*_β_(*n*) profile (Figure 2C, *P* < 0.001 with FET, *k* = 0.620 with CKT and Supplementary Figure S2C for *L_β_* ≥ 1 AA, *P* < 0.001 with FET, *k* = 0.764 with CKT). A very good agreement was also found between the *F*_β_(*n*) profile and the TANGO score profile [16], as shown in Figure 2D. TANGO predicted the two regions 16–22 and 29–41 (*TANGO score > 0*), in substantial agreement with the regions 12–21 and 31–42 obtained with *L_β_* ≥ 4 AA (*F*_β_(*n*) ≥ 40%) in the experimental *F*_β_(*n*) profile (Figure 2D, *P* <0.001 with FET, *k* = 0.620 with CKT and Supplementary Figure S2D for *L_β_* ≥ 1 AA, *P* < 0.001 with FET, *k* = 0.764 with CKT).

We then tested WALTZ, which predicts the two regions 15–23 and 26–42 (*WALTZ score > 75%*), in good agreement with the regions 12–21 and 31–42 obtained with *L_β_* ≥ 4 AA (*F*_β_(*n*) ≥ 40%) in the experimental *F*_β_(*n*) profile (Figure 2E, *P* < 0.001 with FET, *k* = 0.518 with CKT and Supplementary Figure S2E for *L_β_* ≥ 1 AA, *P* < 0.001 with FET, *k* = 0.751 with CKT). We finally tested the second version of PASTA (PASTA 2.0), which makes predictions based upon a dataset containing structures of natural β-sheet proteins and a statistical energy function to assess amyloid aggregation [17]. The algorithm predicts a major, extended peak encompassing residues 12–42 (–*∆G_agg_* > 5 units), which include both the first and second peaks 12–21 and 31–42 obtained with *L_β_* ≥ 4 AA in the experimental *F*_β_(*n*) profile, although the interruption between them remains undetected by the algorithm (Figure 2F and Supplementary Figure S2F). Based on statistical analysis, the agreement is good (Figure 2F, *P* < 0.001 with FET, *k* = 0.561 with CKT and Supplementary Figure S2F for *L_β_* ≥ 1 AA, *P* < 0.001 with FET, *k* = 0.686 with CKT).

### Agreement between experimental structure-based profile and predicted sequence-based aggregation propensity profiles for αS

The same analysis was repeated on a more complex system: the 140-residue long αS protein. For this protein, 83 amyloid fibril or protofilament structures have been deposited in the PDB, all listed in the *Amyloid Atlas*. The larger number of structures (almost three-fold higher than those of Aβ) makes the statistical analysis even more robust. The *Amyloid Atlas* structures are actually 89, but we excluded six structures with large sequence deletions or insertions: two structures were either N- or C-terminally truncated by over 35 residues (PDB entries 7lc9, 6osm) and four structures had significant insertions (PDB entries 8bqv, 8bqw, 8ce7, 8ceb). 69 and 14 structures correspond to fibrils obtained with wild-type peptides or mutated/modified forms, respectively. Only two structures were determined with ssNMR, with the remainder 81 solved with cryoEM (Supplementary Tables S3 and S4). The 83 structures in the dataset were determined by 20 different research groups, in all cases after 2015 (Supplementary Tables S3 and S4).

**Figure 3:**
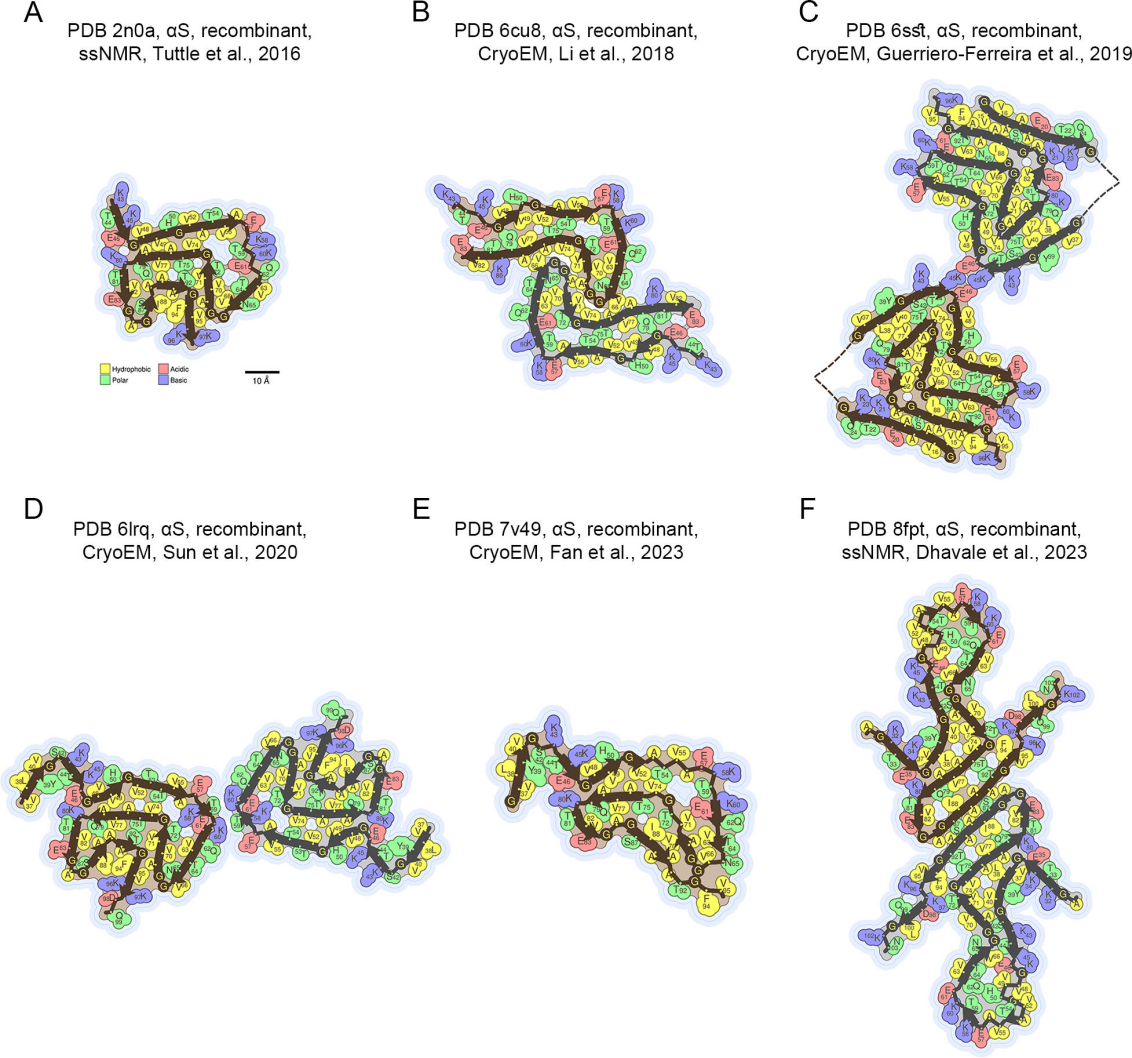
Representative amyloid fibril/protofilament structures of αS deposited in the PDB and listed in the *Amyloid Atlas* [21]. (**A-F**) The colour code and size bar reported in Panel A refer to all 6six Panels. In each case, the PDB entry, protein length, origin of analyzsed fibrils, technique and reference are reported. Each structure reports the residues adopting a β-sheet conformation. Other details as in Figure 1 legend.

Figure 3 reports six representative αS amyloid fibril structures. Similarly to the Aβ case, the various αS structures were very diverse. By comparing the β-strand segments in these structures, analysed individually one by one, with those resulting from the ZYGGREGATOR aggregation propensity profile [13], which identified the same stretches as residues 38–39, 50–53, 65–76, 87–92 (*Z^prof^*_agg_ ≥ 1), we see again that the agreement ranges from good to poor, as determined with the one-tail FET *P* value and CKT *κ* value (Supplementary Figure S3, Supplementary Table S3). This scenario is similar when using the other algorithms (data not shown). The comparisons were extended to all 83 structures of αS fibrils and to all five algorithms (Supplementary Tables S3 and S4). Box plots showing the distributions of κ values over all the 83 structures and obtained with each of the five algorithms are shown in Supplementary Figure S7C (L*_β_* ≥ 4 AA) and Supplementary Figure S7D (L*_β_* ≥ 1 AA). They range for each of the five algorithms from low to high κ values, although in many cases and on average they are high, indicating that almost all algorithms have good agreements with one or more structures.

**Figure 4:**
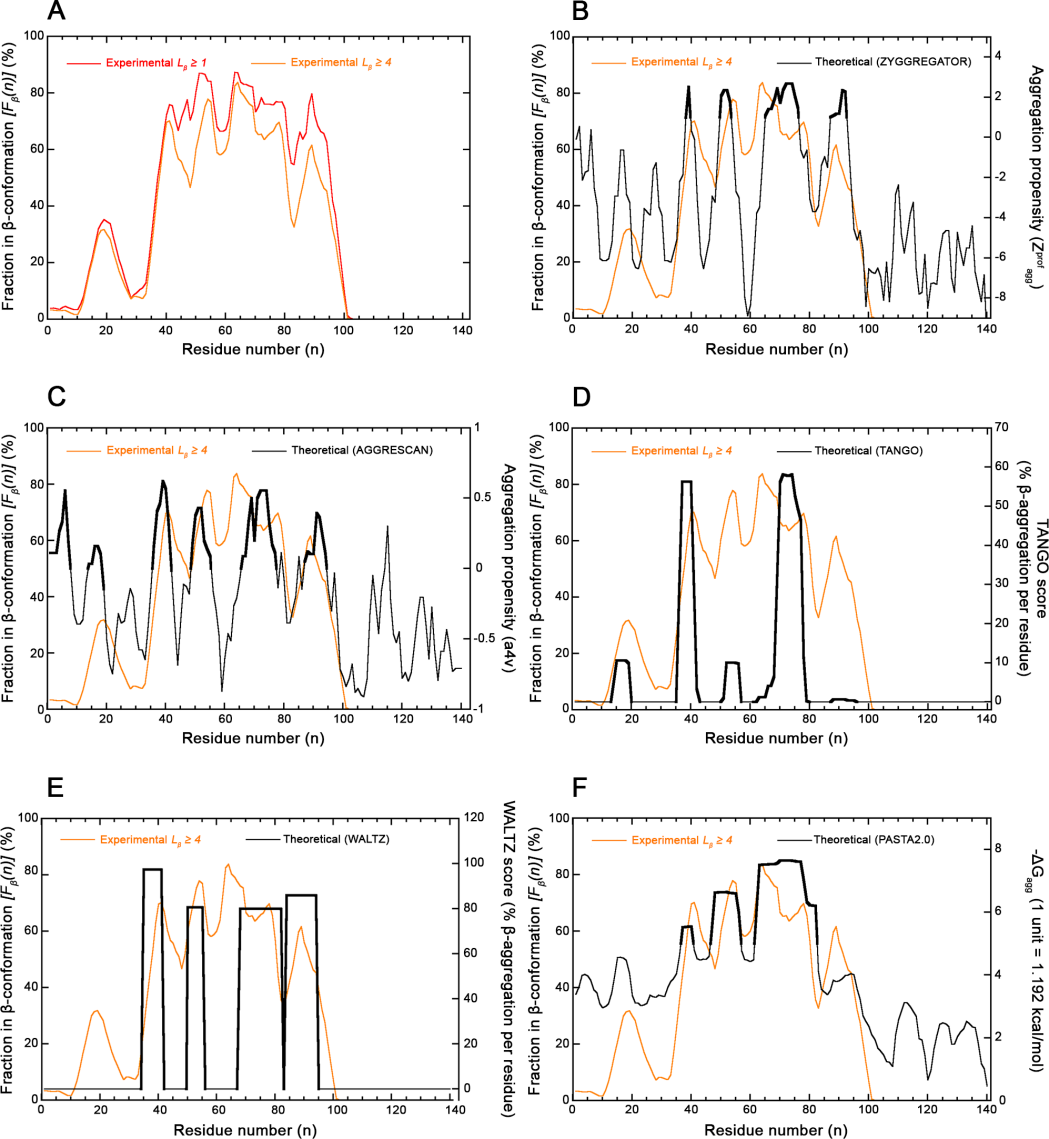
Experimental and theoretical profiles for αS. (**A**) Experimental, structure-based β-sheet preference profile (*F*_β_(*n*)) vs. residue number *n* determined with all 83 available amyloid fibril structures of full-length αS deposited in the PDB and listed in the *Amyloid Atlas* [21]. Profiles were edited by considering residues in the β-sheet conformation when belonging to all β-strands with *L*_β_ ≥ 1 AA (red) and *L*_β_ ≥ 4 AA (orange). (**B-F**) Structure-based *F*_β_(*n*) profile vs. residue number *n* obtained for *L*_β_ ≥ 4 AA (orange) compared with the predicted aggregation propensity profile (black) edited with ZYGGREGATOR (**B**), AGGRESCAN (**C**), TANGO (**D**), WALTZ (**E**) and PASTA (**F**) as described [13,16–19]. In all cases, parameters and units of the experimental structure-based (orange) and predicted algorithm-based (black) profiles are reported on the left and right *Y* axes, respectively. Sequence regions predicted theoretically to adopt a β-strand conformation (*Z^prof^_agg_* ≥ 1, *a4v* > 0, *TANGO score* > 0, *WALTZ score* > 0, *–∆G_agg_* > 5 units, respectively) are highlighted in bold.

The structure-based β-sheet propensity profile of the αS sequence in the amyloid state as a function of residue number *n* (*F*_β_(*n*) *versus n*) shows again that the various regions of the αS sequence have very different β-sheet preferences (Figure 4A). Residues adopting more frequently a β-sheet conformation in the amyloid fibrils are primarily in the large central portion encompassing residues 37–95 (including the hydrophobic region of residues 61–95) [30], with the N- and C-terminal portions of the sequence exhibiting small β-sheet preferences, if any (Figure 4A). In particular, four peaks spanning residues 40–43, 48–56, 61–80 and 88–90 are evident (*F*_β_(*n*) ≥ 70%) when considering β-strands with *L*_β_ ≥ 1 AA (Figure 4A, red line). These become segments 39–43, 50–56, 60–79 and 88–89 (*F*_β_(*n*) ≥ 60%), when considering β-strands with *L*_β_ ≥ 4 AA (Figure 4A, orange line).

These sequence segments correspond, to a very good extent and substantial agreement, to those identified with the ZYGGREGATOR aggregation propensity profile previously reported (*Z^prof^*_agg_ ≥ 1), i.e. 38–39, 50–53, 65–76, 87–92 (Figure 4B, *P* < 0.001 with FET, *k* = 0.568 with CKT and Supplementary Figure S4B for *L_β_* ≥ 1 AA, *P* < 0.001 with FET, *k* = 0.496 with CKT). They also correspond with good agreement to four of the six sequence regions identified by AGGRESCAN (*a4v* ≥ *0*), i.e. 36–42, 49–55, 66–77, 87–94 (Figure 4C, *P* < 0.001 with FET, *k* = 0.433 with CKT and Supplementary Figure S4C for *L_β_* ≥ 1 AA, *P* < 0.001 with FET, *k* = 0.398 with CKT). In this case, the algorithm also predicts the two regions 1–7 and 14–19, which have, however, null or modest *F*_β_(*n*) values (Figure 4C). TANGO predicts the regions 14–19, 36–42, 51–56, 62–79 and 88–95 (*TANGO score > 0*) and all of them correspond to peaks in the experimental *F*_β_(*n*) profile, although the first is modest (Figure 4D). The agreement is very good (Figure 4D, *P* < 0.001 with FET, *k* = 0.667 with CKT and Supplementary Figure S4D for *L_β_* ≥ 1 AA, *P* < 0.001 with FET, *k* = 0.593 with CKT). WALTZ also provides a very good prediction with sequence regions 35–41, 50–55, 68–82 and 84–94 (*WALTZ score > 75%*), in very good agreement with the four regions with high *F*_β_(*n*) values (Figure 4E, *P* < 0.001 with FET, *k* = 0.501 with CKT and Supplementary Figure S4E for *L_β_* ≥ 1 AA, *P* < 0.001 with FET, *k* = 0.467 with CKT). PASTA 2.0 can identify three sequence segments that correspond to the three major peaks in the experimental *F*_β_(*n*) profile, as the algorithm reports the sequence regions 37–40, 47–56 and 62–82 (Figure 4F, *P* < 0.001 with FET, *k* = 0.711 with CKT and Supplementary Figure S4F for *L_β_* ≥ 1 AA, *P* < 0.001 with FET, *k* = 0.743 with CKT).

### Agreement between experimental structure-based profile and predicted sequence-based aggregation propensity profiles for 4R tau

**Figure 5:**
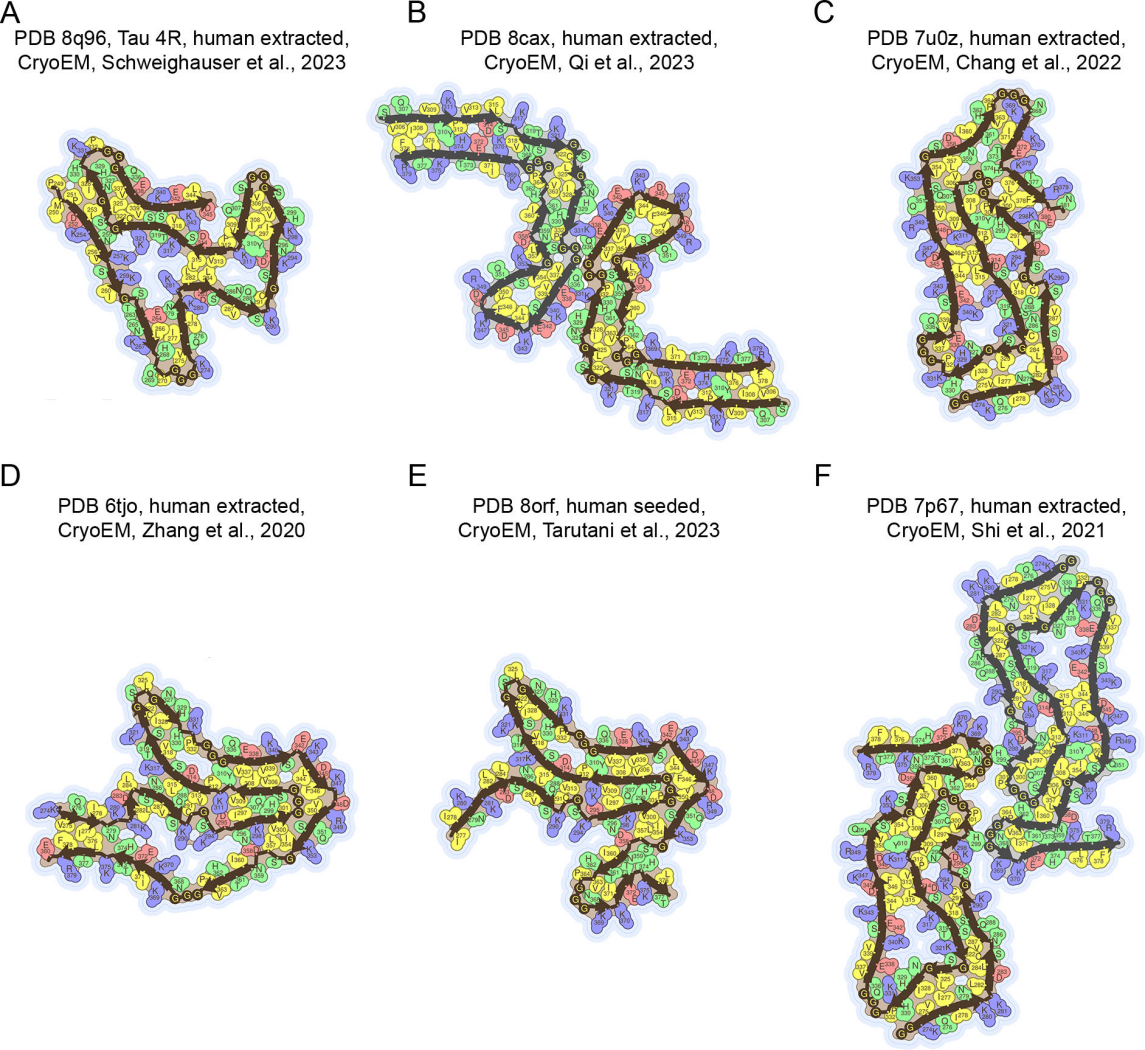
Representative amyloid fibril/protofilament structures of tau (4R isoforms) deposited in the PDB and listed in the *Amyloid Atlas* [21]. (**A-F**) The colour code and size bar reported in Panel A refer to all 6six Ppanels. In each case, the PDB entry, protein length, origin of analyzsed fibrils, technique and reference are reported. Each structure reports the residues adopting a β-sheet conformation. Other details as in Figure 1 legend.

As a further analysis, we evaluated the 383-, 412- and 441-residue long, 4R isoforms of the tau protein, called 0N4R, 1N4R and 2N4R, respectively, for which 24 amyloid structures have been deposited in the PDB and *Amyloid Atlas*, of which 20 are 2N4R, 3 are 1N43 and 1 is 0N4R. We excluded all structures from tau peptides, 3R isoform or mixed 3R/4R isoforms, because the C-terminal repeat domains include β-strand regions and different isoforms are difficult to compare. The 24 structures for the 4R isoform were determined by seven different research groups in nine different papers, in all cases after 2019 (Supplementary Tables S5 and S6). Similar to the Aβ and αS cases, the various 4R tau amyloid fibril structures were very diverse, as shown for six representative structures (Figure 5, Supplementary Figure S5). By comparing the β-strand segments in these structures, analysed individually one by one, with those resulting from the various algorithms, we see that the agreement ranges from scarce to fair, as determined with the one-tail FET *P* value and CKT *κ* value (Supplementary Figures S5 and S7E,F; Supplementary Tables S5 and S6). This underlines again the structural polymorphism existing for the same protein, which appears to increase with protein length and complexity.

**Figure 6:**
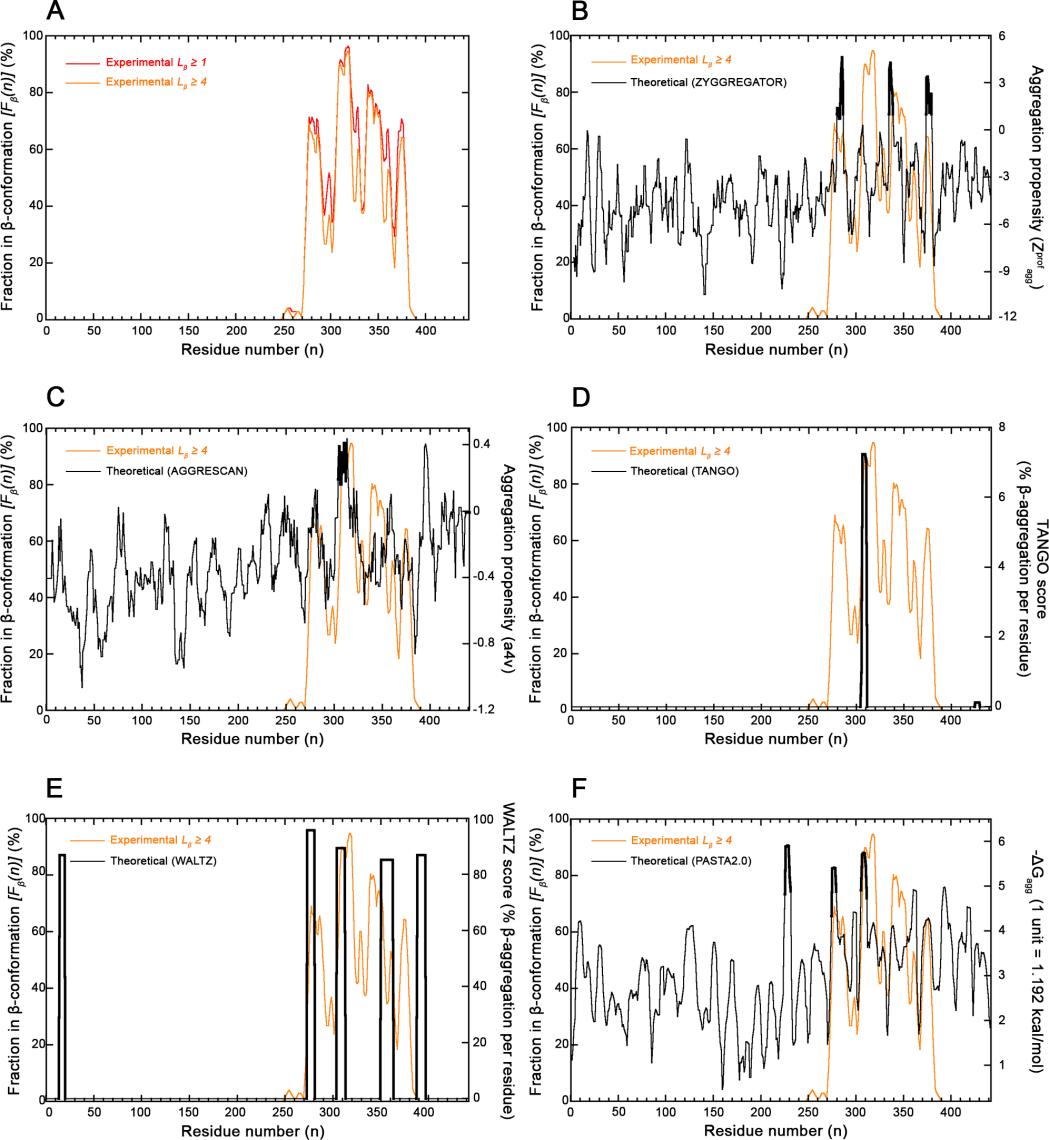
Experimental and theoretical profiles for 4R tau fibrils/protofilaments. (**A**) Experimental, structure-based β-sheet preference profile *(F*_*β*_*(n))* vs. residue number *n* determined with all 24 available amyloid fibril structures of 4R tau deposited in the PDB and listed in the *Amyloid Atlas* [21]. Profiles were edited by considering residues in the β-sheet conformation when belonging to all β-strands with *L*_β_ ≥ 1 AA (red) and *L*_β_ ≥ 4 AA (orange). (**B-F**) Structure-based *F*_*β*_*(n)* profile vs. residue number *n* obtained for *L*_β_ ≥ 4 AA (orange) compared with the predicted aggregation propensity profile (black) edited with ZYGGREGATOR (**B**), AGGRESCAN (**C**), TANGO (**D**), WALTZ (**E**) and PASTA (**F**) as described [13,16–19]. In all cases, parameters and units of the experimental structure-based (orange) and predicted algorithm-based (black) profiles are reported on the left and right *Y* axes, respectively. Sequence regions predicted theoretically to adopt a β-strand conformation (*Z^prof^_agg_* ≥ 1, *a4v* > 0, *TANGO score* > 0, *WALTZ score* > 0, *–∆G_agg_* > 5 units, respectively) are highlighted in bold.

Similar to the αS case, the *F*_β_(*n*) profile of 4R tau shows again that the various sequence regions have very different β-sheet preferences (Figure 6A). Residues adopting more frequently a β-sheet conformation in the amyloid fibrils are primarily in the large portion encompassing residues 270–380 (Figure 6A). In particular, four major peaks spanning residues 277–286, 307–323, 338–352 and 374–376 are evident (*F*_β_(*n*) ≥ 70%) when considering β-strands with *L*_β_ ≥ 1 AA (Figure 6A, red line). These correspond to segments 276–288, 306–321, 337–352 and 373–376 (*F*_β_(*n*) ≥ 60%), when considering β-strands with *L*_β_ ≥ 4 AA (Figure 6A, orange line). Three out of four *F*_β_(*n*) segments correspond, with substantial agreement, to those identified with the ZYGGREGATOR (*Z^prof^*_agg_ ≥ 1) algorithm (Figure 6B). The remaining 306–321 segment of *F*_β_(*n*) corresponds to a peak of *Z^prof^*_agg_ that remains slightly below the threshold of 1 (Figure 6B). The agreement is, however, substantial as indicated by the overlapping of the two very minor peaks around residues 327–328 and 359–360, which remain below the thresholds for both *F*_β_(*n*) and *Z^prof^*_agg_ (Figure 6B). The statistical analysis confirms the agreement for *L_β_* ≥ 4 AA (Figure 6B, *P* < 0.001 with FET, *k* = 0.786 with CKT) and for *L_β_* ≥ 1 AA (Supplementary Figure S6B), *P* < 0.001 with FET, *k* = 0.633 with CKT), as well as for the AGGRESCAN (*a4v* ≥ *0*) method (Figure 6C for *L_β_* ≥ 4 AA, *P* < 0.001 with FET, *k* = 0.622 with CKT and Supplementary Figure S6C for *L_β_* ≥ 1 AA, *P* < 0.001 with FET, *k* = 0.516 with CKT), the WALTZ (*WALTZ score > 75%*) tool (Figure 6E for *L_β_* ≥ 4 AA, *P* < 0.001 with FET, *k* = 0.642 with CKT and Supplementary Figure S6E for *L_β_* ≥ 1 AA, *P* < 0.001 with FET, *k* = 0.632 with CKT) and the PASTA 2.0 tool (Figure 6F for *L_β_* ≥ 4 AA, *P* < 0.001 with FET, *k* = 0.649 with CKT and Supplementary Figure S6F for *L_β_* ≥ 1 AA, *P* < 0.001 with FET, *k* = 0.653 with CKT). Perhaps the least satisfactory agreement was found with TANGO in this case (*TANGO score > 0*), which predicts only one region (Figure 6D, *P* < 0.001 with FET, *k* = 0.418 with CKT and Supplementary Figure S6D for *L_β_* ≥ 1 AA, *P* < 0.001 with FET, *k* = 0.472 with CKT).

Although satisfactory, the full agreement between algorithm- and *F*_β_(*n*)-identified segments is more difficult to achieve for 4R tau than for Aβ and αS, both because of the larger number of residues, and thus higher complexity, of 4R tau, and because of the smaller number of amyloid structures available, which contributes to a statistically less robust *F*_β_(*n*) profile. A stricter comparison awaits the enlargement of the *Amyloid Atlas* database for this specific protein.

## Discussion

We have reported an agreement between the regions of the amino acid sequences of Aβ, αS and 4R tau predicted with each of the five analyzed computational methods to adopt a β-strand conformation in amyloid fibrils and the sequence regions often adopting a β-sheet conformation among amyloid polymorphs, which emerge as peaks in the structure-based β-sheet preference profile named here as the *F*_β_(*n*) profile. For all three proteins, the agreement is very statistically significant in all cases using both the Fisher’s exact test (*P* < 0.001) and the more stringent Cohen’s kappa test (*k* > 0.40). In the latter case, the agreement ranges from good (0.40 < *k* ≤ 0.60) to extremely good (0.80 < *k ≤* 1.00). It is never poor (0.00 < *k* ≤ 0.20). Nor is it even fair (0.20 < *k* ≤ 0.40).

This experimentally driven analysis using all PDB structures available for Aβ/αS/tau comes to similar conclusions as force-field-based analyses of the same structures [31,32], namely that the concept of aggregation-promoting regions still holds in the many and new ssNMR and cryoEM structures. Although the entire sequence is present in the amyloid fibrils, it is the aggregation-promoting regions that actually adopt β-sheet structure with the rest of the polypeptide chain deviating significantly from this ideal.

These results provide insight into the physicochemical principles existing across the wide distributions of polymorphs formed by the same protein, as they show that individual residues and regions of the sequence with most favourable parameters to form a β-sheet structure are most represented in this distribution. The problem of the simultaneous optimisation of the β-sheet propensity, hydrophobicity and charge in the amyloid state has multiple and energetically nearly equivalent solutions in the conformational space of proteins, where many apparently different structures are stable enough to form under well-defined conditions. Polymorphism thus emerges as a fundamental property of the undesired, non-functional amyloid state likely resulting from a lack of evolutionary pressure and therefore yielding a flat free energy landscape. While the native folded state of a protein can be typically represented as a single structure, the amyloid state should be represented as a collection of polymorphs, in which the same basic rules of prevalence of residues with high β-sheet propensity, hydrophobicity and low charge in the fibril core clearly emerge when analysing statistically all of them together.

## Materials and methods

### Construction of the database

The database used in the present work was retrieved from the *Amyloid Atlas* [21], available at https://people.mbi.ucla.edu/sawaya/amyloidatlas/ (URL retrieved on April 8, 2024). We used the version updated to December 12, 2023. The following 30 PDB entries were included in the analysis of Aβ: 2beg 2lmq, 8bfa, 5kk3, 8azt, 8bfz, 7f29, 5oqv, 2lnq, 2lmn, 2lmo, 6ti5, 6ti6, 2lmp, 2mpz, 2m4j, 5aef, 8eze, 6w0o, 6shs, 8bfb, 8ezd, 2mxu, 2nao, 7q4b, 8azs, 7q4m, 8bg0, 8bg9, 2mvx. PDB entries 6oiz and 6nb9 were excluded, as they report fibrils from Aβ fragments. The following 83 PDB entries were included in the analysis of αS: 2n0a, 6a6b, 6cu7, 6cu8, 6h6b, 6l1t, 6l1u, 6l4s, 6lrq, 6osj, 6osl, 6peo, 6pes, 6rt0, 6rtb, 6sst, 6ssx, 6ufr, 6xyo, 6xyp, 6xyq, 7c1d, 7e0f, 7l7h, 7nca, 7ncg, 7nch, 7nci, 7ncj, 7nck, 7ozg, 7ozh, 7uak, 7v47, 7v48, 7v49, 7v4a, 7v4b, 7v4c, 7v4d, 7wmm, 7wnz, 7wo0, 7xjx, 7xo0, 7xo1, 7xo2, 7xo3, 7yk2, 7yk8, 7yng, 7ynl, 7ynm, 7ynn, 7yno, 7ynp, 7ynq, 7ynr, 7yns, 7ynt, 8a4l, 8a9l, 8ads, 8adu, 8adv, 8adw, 8aex, 8cyr, 8cys, 8cyt, 8cyv, 8cyw, 8cyx, 8cyy, 8cz0, 8cz1, 8cz2, 8cz3, 8cz6, 8fpt, 8h03, 8h04, 8h05. Six PDB entries were excluded, as they are formed from αS molecules with truncations of segments longer than 30 consecutive residues (7lc9, 6osm) or with significant insertions (8bqv, 8bqw, 8ce7, 8ceb). The following 24 PDB entries were included in the analysis of 4R tau (all are 2N unless specified otherwise): 8cax, 6tjo, 7p6d, 8orf (1N), 8org (1N), 6tjx, 7p6e, 7p65, 7u0z, 7p66, 7p67, 7p68, 7p6a, 7p6b, 7p6c, 8q96 (0N), 8q92 (1N), 6qjh, 6qjm, 6qjp, 6nwp, 8caq, 8byn, 6nwq. All PDB entries involving 3R tau, mixed 3R/4R tau or shorter fragments were excluded.

In addition, a few PDB entries that contain more than one molecule exhibit discrepancies among the different molecules of the same fibril. This was observed in PDB entries 2lmn, 2lnq, 5kk3, 6ti6, 6w0o, 8bg0, 8bg9 (all containing two molecules) and 2lmq (three molecules) for Aβ, in PDB entries 6pes, 6xyo, 6xyp, 6xyq, 7l7h, 7nca, 7nch, 7ncj, 8aex, 8cyv, 8cyy (all containing two molecules) and 6l1u (three molecules) for αS, and in PDB entry 7p68 (two molecules) for 4R tau. In these cases, we generated separate elements of our database for each molecule, each element describing one of the molecules present in the original PDB file. The final database contained therefore 39 elements for Aβ, 96 elements for αS and 25 elements for 4R tau. Each structural element of the database was analysed manually to identify the residues that assume a β-strand conformation, assigning each residue to a structural class (non-β-strand, β-strand) on the basis of the Ramachandran plot of each structure. The boundaries of the β-strands corresponded exactly to those displayed on the *Amyloid Atlas*. Moreover, since residues Gly37-Gly38 of Aβ possess a high conformational freedom of their φ and ψ angles, we examined the subsequent residues and we considered Gly38 in a β-conformation when it was aligned with a β-sheet starting from Val39. The same approach was applied for residues Gly67-Gly68 and Gly84-Ala85-Gly86 of αS. We repeated the same analysis twice by introducing a minimum threshold of 1 and 4 consecutive residues for a β-strand to be considered as such; in the latter case, amino acid residues were classified as “β” only if the β-strand they belong to is at least four residues long.

### Construction of the *F*_β_(*n*) profile

The *F*_β_(*n*) profile of Aβ, αS or 4R tau was obtained, using a script written with the Perl programming language, as follows: for each residue *n* in the sequence, we calculated the fraction of elements in our database that show the same residue *n* involved in an amyloid β-strand. For example, His6 of Aβ adopts a β-conformation in 6 out of 30 PDB amyloid fibril PDB structures, leading to a *F*_β_(*20*) value of 20%. When the same residue *n* did not populate a β-strand in all the molecules of the same PDB entry, we calculated the fraction of molecules of the aggregate showing the same residue *n* under scrutiny in a β-strand. For example, the PDB entry 5kk3 shows two Aβ molecules, but Val24 assumes a β-strand conformation only in one of these two molecules: consequently, we assigned a value of 0.5 to residue 24 of the PDB entry 5kk3. By contrast, the PDB entry 5aef shows two Aβ molecules with Val24 in a β-strand conformation in both cases, leading to a value of 1.0 for residue 24 in this PDB entry. The *F*_β_(*n*) versus *n* profiles obtained were averaged over sliding windows of seven residues.

### Predicted aggregation propensity profiles

Predicted aggregation propensity profiles of Aβ, αS and 4R tau were obtained with the five different algorithms described above: the first version of ZYGGREGATOR [13], the first version of AGGRESCAN [18], TANGO [16], WALTZ [19] and the second version of PASTA [17]. The ZYGGREGATOR aggregation propensity profile reports the residue-dependent aggregation propensity on an input sequence of interest by averaging the values over 7-residue sliding windows and we used a threshold of Z^prof^_agg_ > 1 to identify the residues with the highest local propensities to aggregate [13]. The AGGRESCAN profile was obtained using the online server (http://bioinf.uab.es/aggrescan/) and entering the FASTA format of the human Aβ_1-42_ sequence (>pdb|1iyt|A DAEFRHDSGYEVHHQKLVFFAEDVGSNKGAIIGLMVGGVVIA), human αS sequence (sp|P37840|SYUA_HUMAN Alpha-synuclein MDVFMKGLSK…EGYQDYEPEA) and human 2N4R tau (>8CAX_1|Microtubule-associated protein tau|Homo sapiens (9606) MAEPRQEFEV…ATLADEVSASLAKQGL) and considering the a4v values, which represent the a3v window average. Residues with the highest local propensities to aggregate were identified as hot spots by the server and represented regions with five or more residues with a4v values greater than 0 (HST≥0) and no prolines [18]. The TANGO profile was obtained using the online server (http://tango.crg.es/) and entering the FASTA format of the human Aβ_1-42_, αS and 4R tau sequence described above. We used a threshold of “TANGO score” > 0 to identify the residues with the highest local propensities to aggregate [16]. The WALTZ profile was obtained using the online server (https://waltz.switchlab.org/) and applying the “high sensitivity” threshold available on the server (75%) to identify the residues with the highest local propensities to aggregate [19]. The PASTA profile was obtained from the online server (http://protein.bio.unipd.it/pasta2/) using the upgraded version of the algorithm (PASTA 2.0) and the quantity “opposite of aggregation free energy” (–*∆G_agg_*). A threshold of *–∆G_agg_* > 5 PASTA energy units (1 unit = 1.192 kcal/mol) was used to identify the residues with the highest local propensities to aggregate, as described [17]. Only X-ray solved structures were used to train PASTA 2.0.

### Statistics

For each possible pair of single experimental structure and theoretical profile, we constructed a contingency table (also known as confusion matrix), i.e. a table that classifies the amino acid residues of the protein sequence (Aβ or αS or 4R tau) in four classes: residues in a β-strand according to both theoretical profile and experimental structure (βte), residues in a β-strand according only to theoretical profile (βt∅), residues in a β-strand only in the experimental structure (β∅e), residues in a β-strand neither in the experimental structure nor theoretical profile (β∅∅). The sum βte+βt∅+β∅e+β∅∅ corresponds to the length of the protein (*l*). Two statistical tests were carried out using the Perl programming language. First, Cohen’s kappa (κ) was calculated using the CKT according to the following formula:


(1) $$\kappa =\frac{2∙\left(\beta te∙\beta \emptyset \emptyset -\beta t\emptyset ∙\beta \emptyset e\right)}{\left(\beta te+\beta t\emptyset \right)∙\left(\beta t\emptyset +\beta \emptyset \emptyset \right)∙\left(\beta te+\beta \emptyset e\right)∙\left(\beta \emptyset e+\beta \emptyset \emptyset \right)}$$

Second, the FET was carried out to calculate the probability p to observe the contingency table under scrutiny if the null hypothesis -i.e. the hypothesis that the algorithm assigns amino acid residues to a β-strand on a purely random basis- were correct:


(2) $$p=\frac{\left(\beta te+\beta \emptyset e\right)!∙\left(\beta t\emptyset +\beta \emptyset \emptyset \right)!∙\left(\beta te+\beta t\emptyset \right)!∙\left(\beta \emptyset e+\beta \emptyset \emptyset \right)!}{l!∙\beta te!∙\beta \emptyset e!∙\beta t\emptyset !∙\beta \emptyset \emptyset !}$$

Then, the probabilities to obtain each of the hypothetical tables with a higher level of agreement between the algorithm and experiment (i.e. hypothetical tables with βte or β∅∅ values higher than the observed values) were also calculated. The final FET *P* is the sum of all these probabilities.

FET *P* and CKT *k* values were also determined using each possible pair of experimental, structure-based *F*_β_(*n*) profile and theoretical, algorithm-based profile. The agreement evaluated using the FET *P* value was considered to be significant (*, *P* < 0.05), highly significant (**, *P* < 0.01) and very highly significant (***, *P* < 0.001), according to widely used statistical laws. The agreement between theory and experiment was also evaluated using the CKT *k* value and judged to be scarce (0.00 < *k* ≤ 0.20), fair (0.20 < *k* ≤ 0.40), good (0.40 < *k* ≤ 0.60), very good or substantial (0.60 < *k* ≤ 0.80) and extremely good or almost perfect (0.80 < *k* ≤ 1.00), according to the classes previously proposed [33].

## Supplementary material

online supplementary figure 1.

online supplementary table 1.

## Data Availability

The datasets used and analysed during the current study are available from the corresponding authors on reasonable request.

## Abbreviations

AA: amino acids
AAt: Amyloid Atlas
AD: Alzheimer’s disease
AL: light chain amyloidosis
ATTR: transthyretin amyloidosis
Aβ: amyloid β
CKT: Cohen’s kappa test
EPR: electron paramagnetic resonance
FET: Fisher’s exact test
GFP: green fluorescent protein
NMR: nuclear magnetic resonance
PD: Parkinson’s disease
PDB: Protein Data Bank
4R tau: 4R isoform of tau protein
cryoEM: cryogenic electron microscopy
ssNMR: solid-state nuclear magnetic resonance
αS: α-synuclein
