# Supplementary material for: Structural commonalities determined by physicochemical principles in the complex polymorphism of the amyloid state of proteins
Source: Biochem J. 2025 Jan 22;482(2):BCJ20240602. doi: 10.1042/BCJ20240602 (PMC12133302; doi:10.1042/BCJ20240602)
Supplement: online supplementary figure 1. [file bcj-482-2-BCJ20240602-s001.pdf]

Supplementary Figures

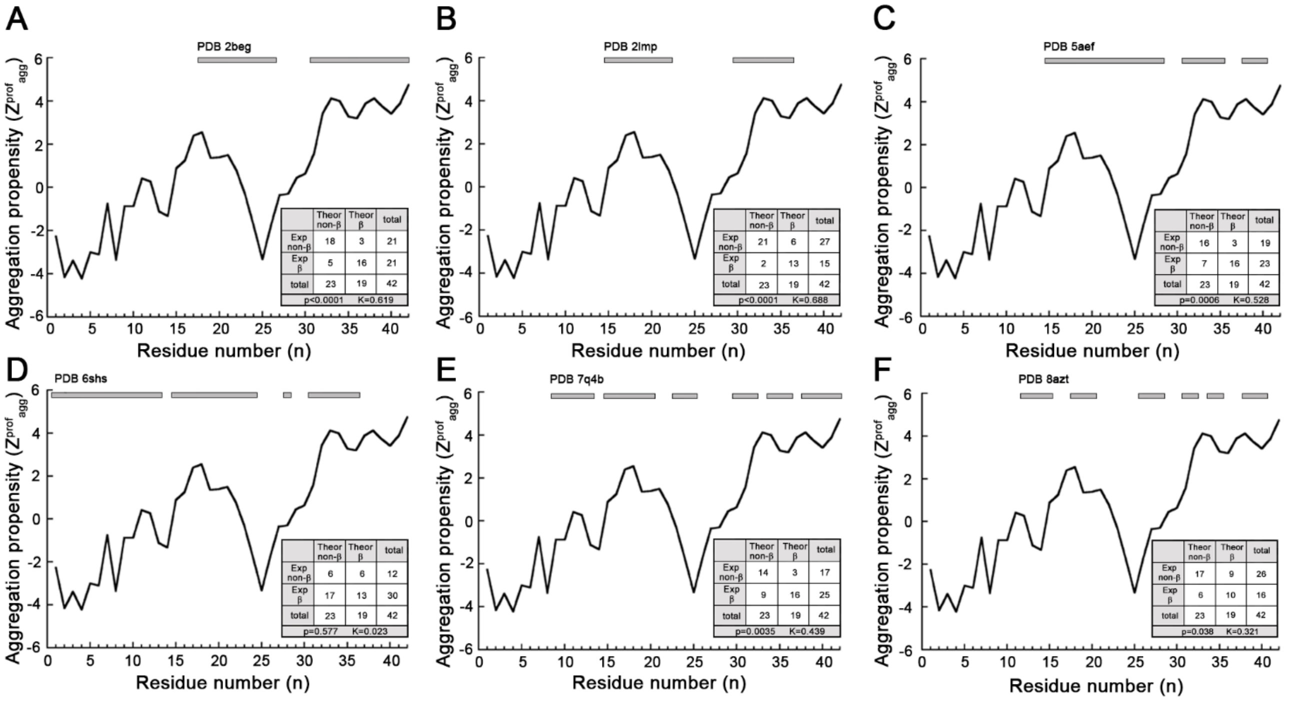

Figure S1. (A-F) Aggregation propensity profile of Aβ_{1-42} edited with ZYGGREGATOR (black line), as previously reported,[1] compared with the residues adopting a β-strand conformation in a fibril structure. Regions of the sequence predicted to adopt a β-strand conformation are those with $Z^{prof}_{agg} \geq 1$. The thick horizontal bars (grey) indicate the residues adopting a β-strand conformation in the corresponding amyloid fibril structure (PDB entry indicated above the bars). Other details as in Figure 1 legend. The inset describes a table with residues adopting a β-sheet or alternative conformation using the experimental and theoretical methods, respectively, as indicated, along with the *p* and *κ* values calculated with the one-tail FET and CKT, respectively.

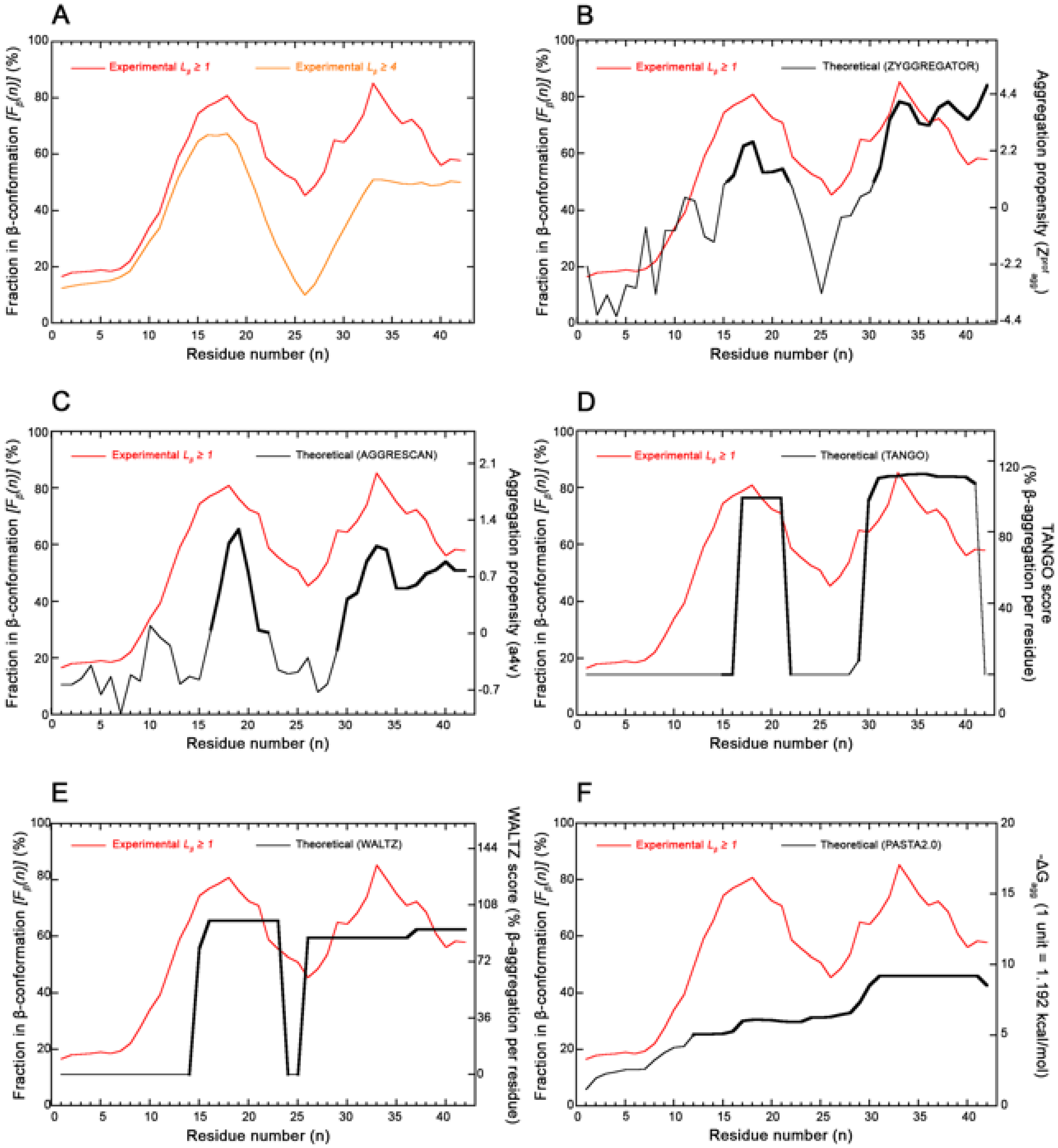

Figure S2. (A) Experimental, structure-based β-sheet propensity profile ($F_\beta(n)$) *vs* residue number n determined with all 30 available amyloid fibril structures of $A\beta_{1-40}$ or $A\beta_{1-42}$ deposited in the PDB and listed in the *Amyloid Atlas*.[2] Profiles were edited by considering residues in the β-sheet conformation when belonging to all β-strands with $L_\beta \geq 1$ AA (red) and $L_\beta \geq 4$ AA (orange). **(B-F)** Structure-based $F_\beta(n)$ profile *vs* residue number n obtained for $L_\beta \geq 1$ AA (red) compared with the predicted aggregation propensity profile (black) edited with ZYGGREGATOR (**B**), AGGRESCAN (**C**), TANGO (**D**), WALTZ (**E**) and PASTA 2.0 (**F**) as described.[1,3–6] In all cases, parameters and units of the experimental structure-based (red) and predicted algorithm-based (black) profiles are reported on the left and right y axes, respectively. Sequence regions predicted theoretically to adopt a β-strand conformation ($Z^{prof}_{agg} \geq 1$, $a4v \geq 0$, *TANGO score* > 0, *WALTZ score* > 75%, $-\Delta G_{agg}$ > 5 units, respectively) are highlighted in bold.

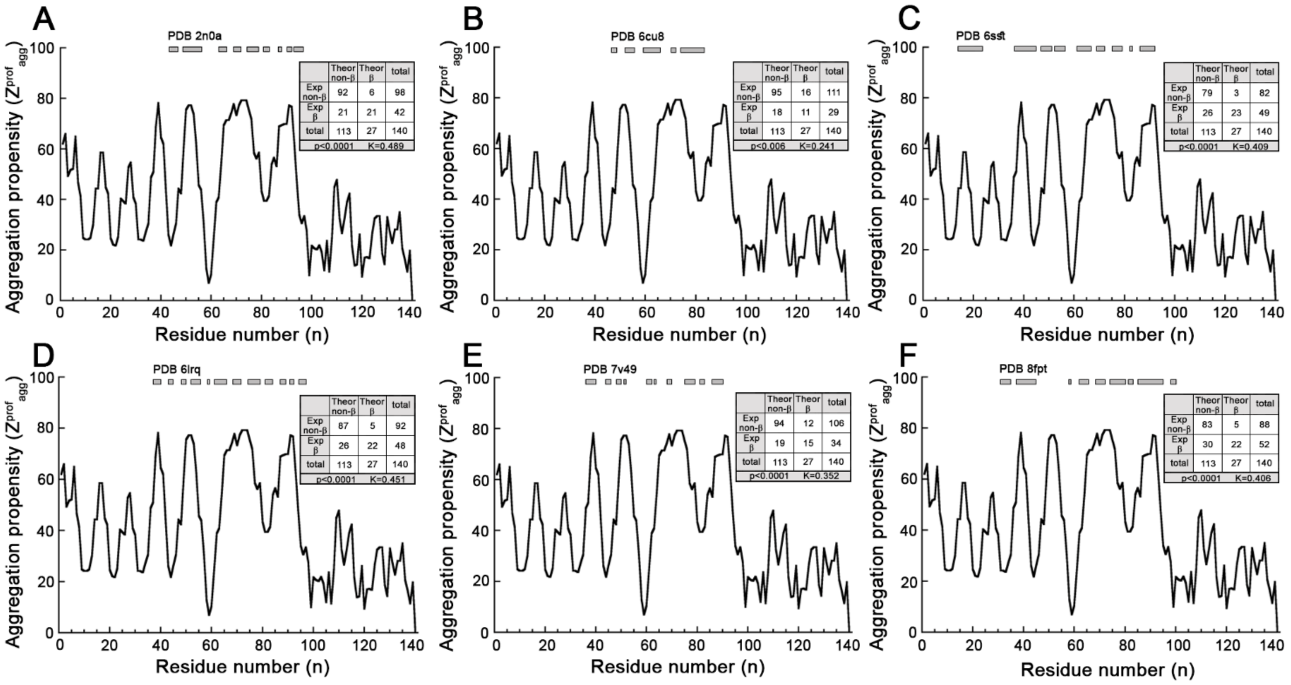

Figure S3. (A-F) Aggregation propensity profile of αS edited with ZYGGREGATOR (black line), as previously reported,[1] in comparison with the residues adopting a β-strand conformation in a fibril structure. Regions of the sequence predicted to adopt a β-strand conformation are those with $Z^{prof}_{agg} \geq 1$. The thick horizontal bars (grey) indicate the residues adopting a β-strand conformation in the corresponding amyloid fibril structure (PDB entry indicated above the bars). Other details as in Figure 1 legend. The inset describes a table with residues adopting a β-sheet or alternative conformation using the experimental and theoretical methods, respectively, as indicated, along with the p and κ values calculated with the one-tail FET and CKT, respectively.

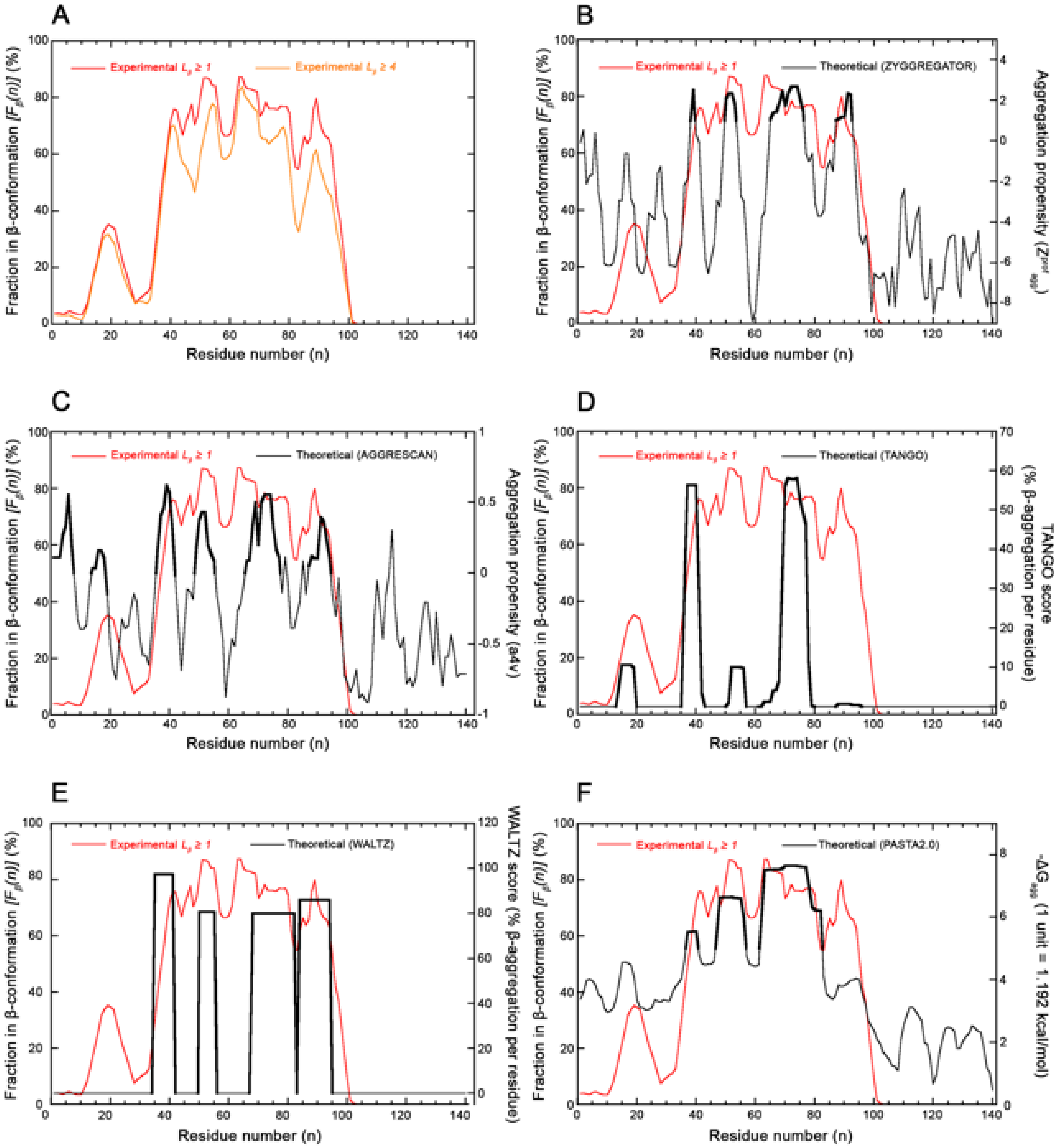

Figure S4. (A) Experimental, structure-based β-sheet preference profile ($F_{\beta}(n)$) *vs* residue number *n* determined with all 83 available amyloid fibril structures of full-length αS deposited in the PDB and listed in the *Amyloid Atlas*.[2] Profiles were edited by considering residues in the β-sheet conformation when belonging to all β-strands with $L_{\beta} \geq 1$ AA (red) and $L_{\beta} \geq 4$ AA (orange). **(B-F)** Structure-based $F_{\beta}(n)$ profile *vs* residue number *n* obtained for $L_{\beta} \geq 1$ AA (red) compared with the predicted aggregation propensity profile (black) edited with ZYGGREGATOR (B), AGGRESCAN (C), TANGO (D), WALTZ (E) and PASTA 2.0 (F) as described.[1,3–6] In all cases, parameters and units of the experimental structure-based (red) and predicted algorithm-based (black) profiles are reported on the left and right *y* axes, respectively. Sequence regions predicted theoretically to adopt a β-strand conformation ($Z^{prof}_{agg} \geq 1$, *a4v* > 0, *TANGO score* > 0, *WALTZ score* > 0, $-\Delta G_{agg} > 5$ units, respectively) are highlighted in bold.

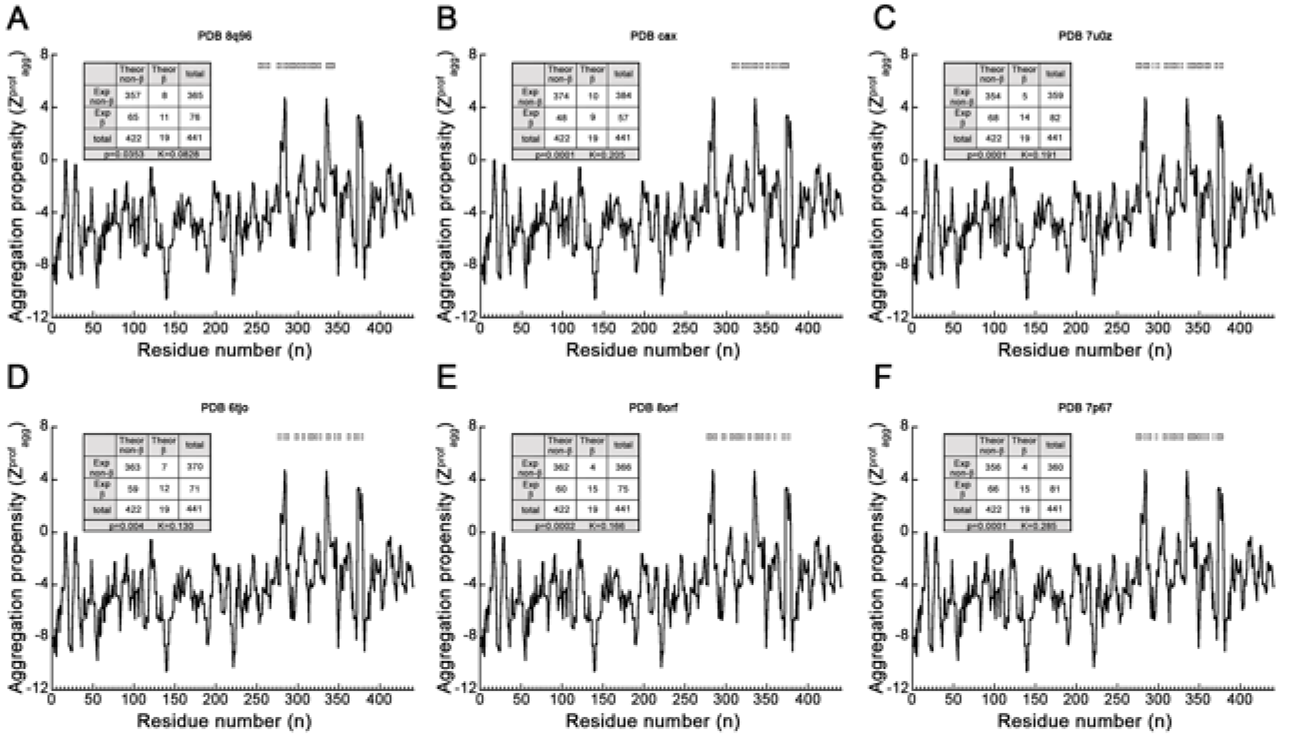

Figure S5. (A-F) Aggregation propensity profile of 4R tau edited with ZYGGREGATOR (black line), as previously reported,[1] in comparison with the residues adopting a β-strand conformation in a fibril structure. Regions of the sequence predicted to adopt a β-strand conformation are those with $Z^{prof}_{agg} \geq 1$. The thick horizontal bars (grey) indicate the residues adopting a β-strand conformation in the corresponding amyloid fibril structure (PDB entry indicated above the bars). Other details as in Figure 1 legend. The inset describes a table with residues adopting a β-sheet or alternative conformation using the experimental and theoretical methods, respectively, as indicated, along with the p and κ values calculated with the one-tail FET and CKT, respectively.

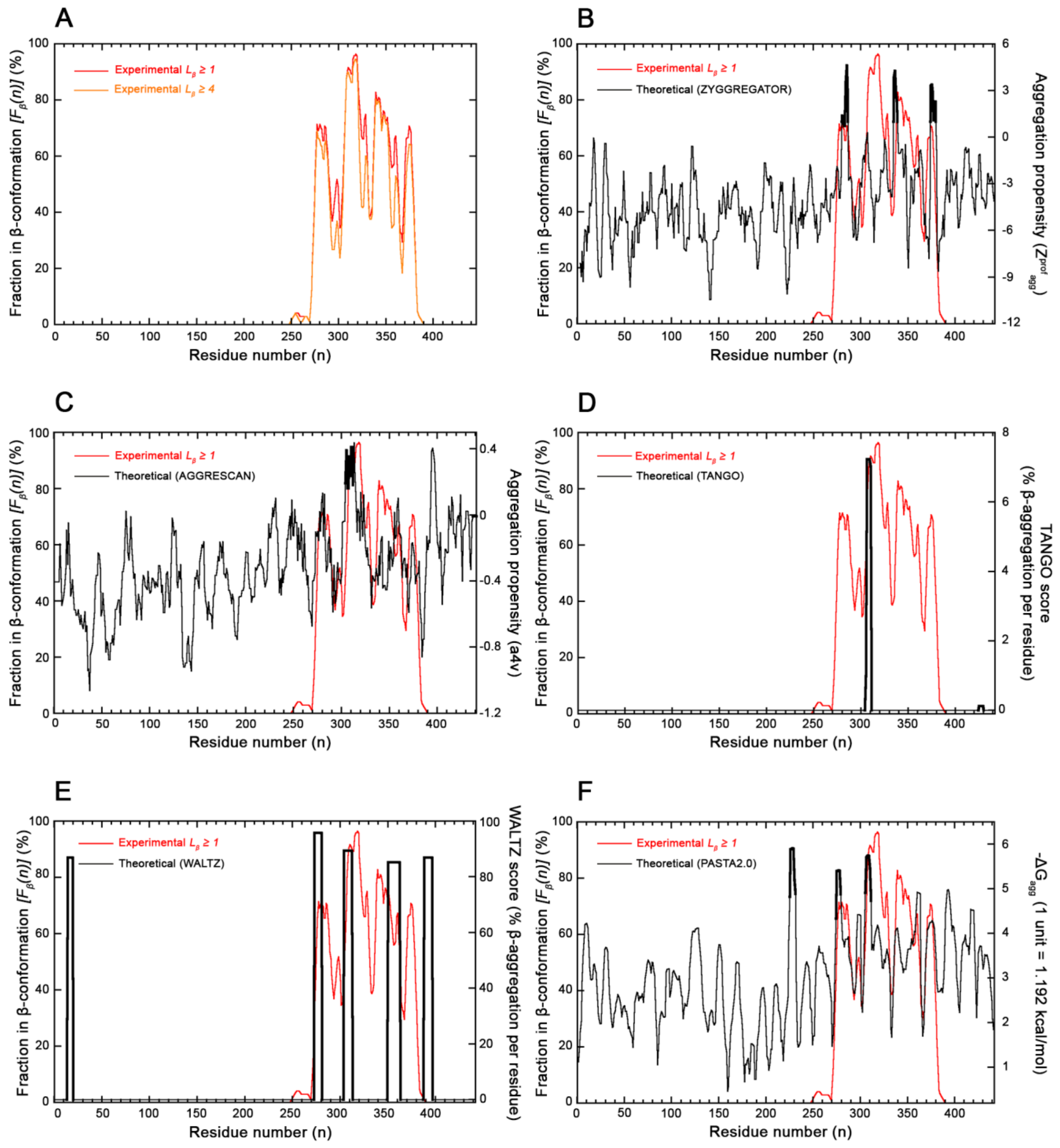

Figure S6. **(A)** Experimental, structure-based β-sheet preference profile ($F_β(n)$) *vs* residue number *n* determined with all 24 available amyloid fibril structures of 4R tau deposited in the PDB and listed in the *Amyloid Atlas*.[2] Profiles were edited by considering residues in the β-sheet conformation when belonging to all β-strands with $L_β \geq 1$ AA (red) and $L_β \geq 4$ AA (orange). **(B-F)** Structure-based $F_β(n)$ profile *vs* residue number *n* obtained for $L_β \geq 1$ AA (red) compared with the predicted aggregation propensity profile (black) edited with ZYGGREGATOR (B), AGGRESCAN (C), TANGO (D), WALTZ (E) and PASTA 2.0 (F) as described.[1,3–6] In all cases, parameters and units of the experimental structure-based (red) and predicted algorithm-based (black) profiles are reported on the left and right *y* axes, respectively. Sequence regions predicted theoretically to adopt a β-strand conformation ($Z^{prof}_{agg} \geq 1$, $a4v > 0$, *TANGO score* > 0, *WALTZ score* > 0, $-\Delta G_{agg} > 5$ units, respectively) are highlighted in bold.

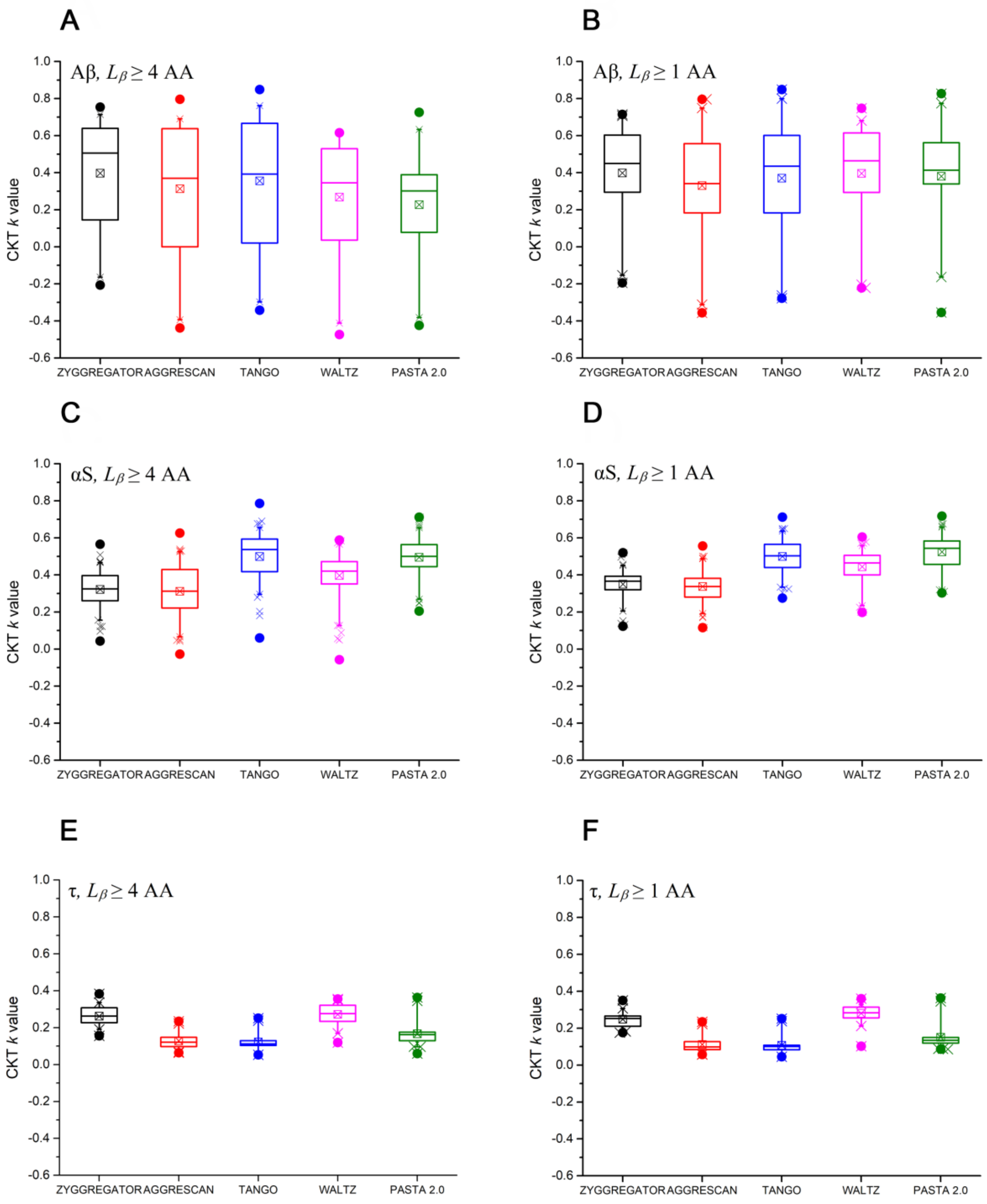

Figure S7. Box plots showing the distributions of the κ values (CKT) obtained when assessing the agreement between the β-strands predicted by the indicated algorithms and the β-strands observed in the individual PDB structures of Aβ (A and B), αS (C and D) or 4R tau (E and F). β-strands in the PDB fibril structures were defined with $L_\beta \geq 4$ AA (A, C and E) and $L_\beta \geq 1$ AA (B, D and F) residues. Each panel shows box plots for ZYGGREGATOR (black), AGGRESCAN (red), TANGO (blue), WALTZ (pink) and PASTA 2.0 (green). The boxes represent the distributions from 25% to 75%. Horizontal lines and crossed squares indicate median and mean values, respectively. Whiskers indicate the 5% and 95% limits. Asterisks indicate outliers and circles correspond to 1% and 99%.

Supplementary References

1. Pawar, A. P. *et al.* Prediction of "Aggregation-prone" and "Aggregation-susceptible" Regions in Proteins Associated with Neurodegenerative Diseases. *J Mol Biol* **350**, 379–392 (2005).
2. Sawaya, M. R., Hughes, M. P., Rodriguez, J. A., Riek, R. & Eisenberg, D. S. The expanding amyloid family: Structure, stability, function, and pathogenesis. *Cell* **184**, 4857–4873 (2021).
3. Conchillo-Solé, O. *et al.* AGGRESCAN: a server for the prediction and evaluation of 'hot spots' of aggregation in polypeptides. *BMC Bioinformatics* **8**, 65 (2007).
4. Fernandez-Escamilla, A.-M., Rousseau, F., Schymkowitz, J. & Serrano, L. Prediction of sequence-dependent and mutational effects on the aggregation of peptides and proteins. *Nat Biotechnol* **22**, 1302–1306 (2004).
5. Maurer-Stroh, S. *et al.* Exploring the sequence determinants of amyloid structure using position-specific scoring matrices. *Nat Methods* **7**, 237–242 (2010).
6. Walsh, I., Seno, F., Tosatto, S. C. E. & Trovato, A. PASTA 2.0: an improved server for protein aggregation prediction. *Nucleic Acids Res* **42**, W301–W307 (2014).
