# Supplementary material for: Structural commonalities determined by physicochemical principles in the complex polymorphism of the amyloid state of proteins
Source: Biochem J. 2025 Jan 22;482(2):BCJ20240602. doi: 10.1042/BCJ20240602 (PMC12133302; doi:10.1042/BCJ20240602)
Supplement: online supplementary table 1. [file bcj-482-2-BCJ20240602-s002.pdf]

Table S1. Amyloid fibril/protofilament structures of Aβ <u>considering L ≥ 4</u> deposited in the PDB and listed in the *Amyloid Atlas*.[1]

PDB ID	Peptide	Fibril origin	Method	***p*** **(one-tail Fisher's exact test)**					***κ*** **(Cohen's kappa coefficient test)**				
				**ZYGGR.**	**AGGRES.**	**TANGO**	**WALTZ**	**PASTA2.0**	**ZYGGR.**	**AGGRES.**	**TANGO**	**WALTZ**	**PASTA2.0**
2beg [2]	$A\beta_{42}$	recombinant	ss-NMR	6.35e-5	1.60e-5	2.53e-4	1.54e-4	8.24e-5	0.619	0.667	0.571	0.571	0.524
2lnq [3]	$A\beta_{40}$	recombinant	ss-NMR	6.98e-5<br>6.98e-5	8.12e-6<br>2.86e-7	5.01e-7<br>1.54e-8	8.45e-5<br>1.17e-5	3.34e-3<br>1.08e-3	0.634<br>0.639	0.691<br>0.796	0.746<br>0.848	0.528<br>0.615	0.339<br>0.404
2lmn [4]	$A\beta_{40}$	recombinant	ss-NMR	6.98e-5<br>1.54e-5	8.12e-6<br>5.09e-5	5.01e-7<br>6.17e-6	8.45e-5<br>3.25e-5	3.34e-3<br>1.93e-3	0.634<br>0.688	0.691<br>0.641	0.746<br>0.696	0.528<br>0.571	0.339<br>0.371
2lmo [4]	$A\beta_{40}$	recombinant	ss-NMR	6.98e-5	8.12e-6	5.01e-7	8.45e-5	3.34e-3	0.634	0.691	0.746	0.528	0.339
6ti5 [5]	$A\beta_{40}$	recombinant	ss-NMR	2.97e-7	1.05e-4	3.17e-5	2.21e-4	1.38e-5	0.754	0.604	0.652	0.592	0.634
6ti6 [5]	$A\beta_{40}/A\beta_{42}$<br>co-fibril	human,<br>recombinant	ss-NMR	3.90e-6<br>2.88e-6	5.00e-5<br>1.60e-5	1.67e-5<br>5.52e-7	3.19e-4<br>1.54e-4	1.89e-6<br>8.24e-5	0.660<br>0.714	0.608<br>0.667	0.653<br>0.762	0.583<br>0.571	0.725<br>0.524
2lmp [4]	$A\beta_{40}$	recombinant	ss-NMR	1.54e-5	5.09e-5	6.17e-6	3.25e-5	1.93e-3	0.688	0.641	0.696	0.571	0.371
2lmq [4]	$A\beta_{40}$	recombinant	ss-NMR	1.18e-5<br>2.85e-4<br>6.98e-5	3.39e-5<br>5.09e-5<br>2.21e-4	2.25e-6<br>6.17e-6<br>4.02e-5	2.07e-4<br>3.25e-5<br>1.17e-5	5.64e-3<br>1.93e-3<br>1.08e-3	0.683<br>0.584<br>0.639	0.637<br>0.641<br>0.592	0.695<br>0.696<br>0.646	0.486<br>0.571<br>0.615	0.309<br>0.371<br>0.404
2mpz [6]	$A\beta_{40}$	recombinant	ss-NMR	9.42e-4	2.21e-4	4.02e-5	3.45e-4	1.08e-3	0.536	0.592	0.646	0.519	0.404
2m4j [7]	$A\beta_{40}$	recombinant,<br>seeded	ss-NMR	0.463	0.472	0.592	0.399	0.279	0.066	-0.064	0.021	-0.071	0.094
5aef [8]	$A\beta_{42}$	recombinant	Cryo-EM	1.74e-5	8.67e-4	7.25e-5	3.86e-4	1.65e-4	0.665	0.523	0.618	0.529	0.488
8eze [9]	$A\beta_{42}$	recombinant,<br>seeded	Cryo-EM	2.88e-6	2.53e-4	1.60e-5	1.78e-3	8.24e-5	0.714	0.571	0.667	0.476	0.524
6w0o [10]	$A\beta_{40}$	seeded	Cryo-EM	5.82e-3<br>3.01e-3	0.065<br>0.035	0.015<br>0.013	9.56e-3<br>4.59e-2	5.58e-2<br>1.93e-3	0.396<br>0.481	0.255<br>0.333	0.330<br>0.392	0.286<br>0.286	0.173<br>0.371
6shs [11]	$A\beta_{40}$	human,<br>extracted	Cryo-EM	0.244	0.592	0.587	0.120	5.64e-3	0.145	-0.014	0.017	-0.237	-0.424

8bfa [12]	$A\beta_{42}$	human, extracted	Cryo-EM	0.152	4.97e-3	0.027	1.29e-3	3.39e-3	-0.207	-0.438	-0.342	-0.474	-0.382
8bfb [12]	$A\beta_{42}$	human, extracted	Cryo-EM	0.227	0.011	0.050	4.45e-3	1.49e-2	-0.164	-0.394	-0.297	-0.412	-0.310
8ezd [9]	$A\beta_{42}$	recombinant, seeded	Cryo-EM	0.355	0.055	0.055	1.80e-3	1.98e-2	0.102	0.269	0.269	0.358	0.224
2mxu [13]	$A\beta_{42}$	recombinant	ss-NMR	0.039	0.063	0.191	0.212	3.26e-2	0.315	0.276	0.179	0.153	0.246
5kk3 [14]	$A\beta_{42}$	recombinant	ss-NMR	2.53e-4<br>2.53e-4	5.83e-4<br>5.83e-4	3.30e-5<br>3.30e-5	1.83e-4<br>1.83e-4	5.02e-3<br>5.02e-3	0.557<br>0.557	0.516<br>0.516	0.613<br>0.613	0.471<br>0.471	0.301<br>0.301
2nao [15]	$A\beta_{42}$	recombinant	ss-NMR	0.035	0.659	0.267	0.134	0.281	0.226	0.009	0.108	0.122	0.072
7q4b [16]	$A\beta_{42}$	human, extracted	Cryo-EM	0.039	0.409	0.409	0.447	0.384	0.315	0.083	0.083	0.064	0.078
8azs [17]	$A\beta_{42}$	human, extracted	Cryo-EM	0.039	0.409	0.409	0.447	0.384	0.315	0.083	0.083	0.064	0.078
7q4m [16]	$A\beta_{42}$	human, extracted	Cryo-EM	1.03e-3	2.48e-3	0.016	0.025	1.04e-3	0.515	0.471	0.375	0.317	0.389
8azt [17]	$A\beta_{42}$	human, extracted	Cryo-EM	0.380	0.065	0.065	0.130	0.281	-0.084	-0.189	-0.189	-0.118	0.072
8bfz [18]	$A\beta_{42}$	human, extracted	Cryo-EM	0.140	0.435	0.565	0.529	4.52e-2	0.194	0.070	-0.028	0.036	0.176
8bg0 [18]	$A\beta_{40}$	human, extracted	Cryo-EM	1.000<br>0.009	1.000<br>0.402	1.000<br>0.141	1.000<br>0.065	1.000<br>0.180	0.000<br>0.324	0.000<br>0.081	0.000<br>0.169	0.00<br>0.174	0.000<br>0.103
8bg9 [18]	$A\beta_{40}$	human, extracted	Cryo-EM	0.100<br>0.276	0.636<br>0.377	0.431<br>0.578	0.475<br>0.467	3.66e-2<br>0.349	0.237<br>0.142	-0.005<br>-0.099	0.075<br>-0.023	0.054<br>-0.055	0.198<br>0.085
7f29 [19]	$A\beta_{42}$	synthetic, glycos. Y10	Cryo-EM	7.94e-4	0.013	1.63e-3	6.46e-3	8.08e-3	0.506	0.370	0.467	0.345	0.274
2mvx [20]	$A\beta_{40}$	recombinant	ss-NMR	0.393	0.252	0.370	0.110	0.183	0.093	-0.156	-0.103	-0.238	-0.174
5oqv [21]	$A\beta_{42}$	recombinant	Cryo-EM	0.534	0.594	0.594	0.649	0.312	-0.034	-0.018	-0.018	-0.005	-0.148

Table S2. Amyloid fibril/protofilament structures of Aβ considering $L \geq 1$ deposited in the PDB and listed in the *Amyloid Atlas*.[1]

PDB ID	Peptide	Fibril origin	Method	*p* (one-tail Fisher's exact test)					*κ* (Cohen's kappa coefficient test)				
				ZYGGR.	AGGRES.	TANGO	WALTZ	PASTA2.0	ZYGGR.	AGGRES.	TANGO	WALTZ	PASTA2.0
2beg [2]	$A\beta_{42}$	recombinant	ss-NMR	6.35e-5	1.60e-5	2.53e-4	1.54e-4	8.24e-5	0.619	0.667	0.571	0.571	0.524
2lnq [3]	$A\beta_{40}$	recombinant	ss-NMR	6.98e-5<br>6.98e-5	8.12e-6<br>2.86e-6	5.01e-7<br>1.54e-8	8.45e-5<br>1.17e-5	3.34e-3<br>1.08e-3	0.634<br>0.639	0.691<br>0.796	0.746<br>0.848	0.528<br>0.615	0.339<br>0.404
2lmn [4]	$A\beta_{40}$	recombinant	ss-NMR	2.60e-3<br>1.54e-5	7.62e-4<br>5.09e-5	1.84e-4<br>6.17e-6	0.014<br>3.25e-5	5.85e-4<br>1.93e-3	0.488<br>0.688	0.543<br>0.641	0.597<br>0.696	0.369<br>0.571	0.438<br>0.371
2lmo [4]	$A\beta_{40}$	recombinant	ss-NMR	2.85e-4	5.09e-5	6.17e-6	8.13e-4	1.93e-3	0.584	0.641	0.696	0.476	0.371
6ti5 [5]	$A\beta_{40}$	recombinant	ss-NMR	1.14e-6	3.09e-4	1.09e-4	6.98e-5	5.35e-6	0.707	0.557	0.603	0.639	0.678
6ti6 [5]	$A\beta_{40}/A\beta_{42}$ co-fibril	human, recombinant	ss-NMR	3.52e-5<br>2.88e-6	4.00e-4<br>1.60e-5	1.72e-4<br>5.52e-7	2.16e-5<br>1.54e-4	1.57e-7<br>8.24e-5	0.569<br>0.714	0.515<br>0.667	0.557<br>0.762	0.681<br>0.571	0.827<br>0.524
2lmp [4]	$A\beta_{40}$	recombinant	ss-NMR	1.54e-5	5.09e-5	6.17e-6	3.25e-5	1.93e-3	0.688	0.641	0.696	0.571	0.371
2lmq [4]	$A\beta_{40}$	recombinant	ss-NMR	6.98e-5<br>2.01e-3<br>4.77e-3	1.93e-4<br>5.53e-3<br>0.012	2.51e-5<br>1.99e-3<br>5.19e-3	1.81e-3<br>3.47e-3<br>0.011	3.34e-3<br>3.05e-3<br>1.55e-2	0.634<br>0.496<br>0.450	0.588<br>0.447<br>0.400	0.645<br>0.499<br>0.450	0.434<br>0.455<br>0.400	0.339<br>0.413<br>0.350
2mpz [6]	$A\beta_{40}$	recombinant	ss-NMR	9.40e-6	1.83e-6	2.77e-7	1.67e-5	1.53e-4	0.698	0.748	0.799	0.653	0.511
2m4j [7]	$A\beta_{40}$	recombinant, seeded	ss-NMR	0.466	0.564	0.403	0.628	0.329	0.065	0.026	0.089	0.000	0.101
5aef [8]	$A\beta_{42}$	recombinant	Cryo-EM	5.72e-4	0.013	2.05e-3	1.82e-5	1.77e-5	0.528	0.384	0.479	0.659	0.601
8eze [9]	$A\beta_{42}$	recombinant, seeded	Cryo-EM	8.97e-4	0.023	3.79e-3	1.90e-3	8.69e-4	0.488	0.341	0.435	0.495	0.517
6w0o [10]	$A\beta_{40}$	seeded	Cryo-EM	1.09e-4<br>3.58e-4	4.95e-4<br>2.04e-3	1.58e-4<br>9.21e-4	1.31e-6<br>9.55e-5	3.27e-5<br>5.90e-7	0.603<br>0.518	0.552<br>0.463	0.601<br>0.506	0.747<br>0.632	0.591<br>0.775
6shs [11]	$A\beta_{40}$	human, extracted	Cryo-EM	0.577	0.229	0.292	0.131	2.36e-2	0.023	-0.143	-0.122	-0.222	-0.355

8bfa [12]	$A\beta_{42}$	human, extracted	Cryo-EM	0.139	0.013	0.061	0.159	0.251	-0.195	-0.356	-0.263	-0.204	-0.163
8bfb [12]	$A\beta_{42}$	human, extracted	Cryo-EM	0.221	0.031	0.115	0.290	0.459	-0.154	-0.313	-0.219	-0.138	-0.075
8ezd [9]	$A\beta_{42}$	recombinant, seeded	Cryo-EM	0.013	0.030	5.91e-3	5.58e-5	3.92e-5	0.384	0.335	0.430	0.615	0.561
2mxu [13]	$A\beta_{42}$	recombinant	ss-NMR	0.010	0.098	0.098	9.42e-3	2.45e-4	0.389	0.243	0.243	0.408	0.540
5kk3 [14]	$A\beta_{42}$	recombinant	ss-NMR	9.57e-4<br>0.013	2.56e-3<br>0.030	2.71e-4<br>5.91e-3	1.47e-6<br>5.58e-5	3.16e-4<br>3.92e-5	0.519<br>0.384	0.474<br>0.335	0.569<br>0.430	0.674<br>0.615	0.453<br>0.561
2nao [15]	$A\beta_{42}$	recombinant	ss-NMR	1.51e-3	0.022	2.64e-3	3.61e-3	0.030	0.449	0.316	0.414	0.323	0.200
7q4b [16]	$A\beta_{42}$	human, extracted	Cryo-EM	3.46e-3	0.050	0.050	0.095	1.40e-2	0.439	0.292	0.292	0.252	0.372
8azs [17]	$A\beta_{42}$	human, extracted	Cryo-EM	8.97e-4	0.023	0.023	0.058	8.74e-3	0.488	0.341	0.341	0.293	0.409
7q4m [16]	$A\beta_{42}$	human, extracted	Cryo-EM	3.46e-3	0.011	0.050	5.04e-4	2.89e-6	0.439	0.387	0.292	0.551	0.686
8azt [17]	$A\beta_{42}$	human, extracted	Cryo-EM	0.038	0.188	0.188	0.025	1.04e-3	0.321	0.183	0.183	0.317	0.389
8bfz [18]	$A\beta_{42}$	human, extracted	Cryo-EM	0.026	0.056	0.169	2.99e-3	1.77e-5	0.339	0.289	0.194	0.464	0.601
8bg0 [18]	$A\beta_{40}$	human, extracted	Cryo-EM	0.060<br>0.181	0.107<br>0.512	0.175<br>0.382	3.47e-3<br>0.022	1.53e-4<br>3.05e-3	0.295<br>0.194	0.246<br>0.045	0.198<br>0.098	0.455<br>0.356	0.511<br>0.413
8bg9 [18]	$A\beta_{40}$	human, extracted	Cryo-EM	0.466<br>0.252	0.125<br>0.036	0.184<br>0.058	0.628<br>0.318	0.387<br>0.486	-0.060<br>-0.140	-0.220<br>-0.304	-0.185<br>-0.278	0.000<br>-0.129	0.099<br>-0.068
7f29 [19]	$A\beta_{42}$	synthetic, glycos. Y10	Cryo-EM	7.55e-3	0.023	3.79e-3	1.72e-4	4.86e-5	0.395	0.341	0.435	0.596	0.624
2mvx [20]	$A\beta_{40}$	recombinant	ss-NMR	0.201	0.623	0.578	0.467	0.333	0.157	-0.005	0.022	0.066	0.122
5oqv [21]	$A\beta_{42}$	recombinant	Cryo-EM	0.430	0.380	0.380	0.413	0.497	0.064	0.079	0.079	0.082	-0.082

Table S3. Amyloid fibril/protofilament structures of αS considering L ≥ 4 deposited in the PDB and listed in the *Amyloid Atlas*.[1]

PDB ID	Protein	Fibril origin	Method	*p* (one-tail Fisher's exact test)					*κ* (Cohen's kappa coefficient test)				
				ZYGGR.	AGGRES.	TANGO	WALTZ	PASTA2.0	ZYGGR.	AGGRES.	TANGO	WALTZ	PASTA2.0
2n0a [22]	α-syn	human recombinant	ss-NMR	4.50e-5	1.07e-3	3.51e-8	3.14e-5	9.92e-12	0.365	0.274	0.472	0.366	0.612
6a6b [23]	α-syn	human recombinant	Cryo-EM	0.012	0.093	2.57e-5	7.25e-6	6.99e-7	0.211	0.126	0.358	0.393	0.446
6cu7 [23]	α-syn	human recombinant	Cryo-EM	0.039	0.375	7.81e-5	1.88e-3	1.35e-9	0.170	0.043	0.339	0.266	0.543
6cu8 [23]	α-syn	human recombinant	Cryo-EM	0.055	0.348	6.26e-5	1.44e-3	1.48e-10	0.156	0.047	0.323	0.268	0.570
6h6b [24]	α-syn	human recombinant	Cryo-EM	4.91e-3	0.235	1.81e-4	6.11e-5	7.51e-8	0.266	0.080	0.329	0.367	0.509
6l1t [25]	α-syn	semi-synthetic	Cryo-EM	4.65e-4	3.28e-6	3.91e-8	2.81e-5	1.25e-8	0.215	0.363	0.420	0.309	0.394
6l1u [25]	α-syn	semi-synthetic	Cryo-EM	0.056<br>0.024<br>0.056	1.49e-4<br>1.06e-5<br>1.49e-4	6.05e-6<br>2.95e-7<br>6.05e-6	1.80e-3<br>2.73e-4<br>1.80e-3	1.11e-10<br>3.43e-12<br>1.11e-10	0.122<br>0.156<br>0.122	0.309<br>0.367<br>0.309	0.366<br>0.425<br>0.366	0.243<br>0.294<br>0.243	0.478<br>0.535<br>0.478
6l4s [26]	α-syn	recombinant	Cryo-EM	6.93e-5	1.83e-3	9.36e-9	6.19e-5	1.35e-15	0.353	0.261	0.493	0.352	0.712
6lrq [27]	α-syn	recombinant	Cryo-EM	8.31e-7	2.69e-4	2.84e-11	2.32e-11	2.19e-13	0.428	0.311	0.573	0.588	0.648
6osj [28]	α-syn	human recombinant	Cryo-EM	9.85e-4	0.056	8.14e-6	2.57e-7	1.50e-8	0.289	0.148	0.380	0.455	0.510
6osl [28]	α-syn	human recombinant	Cryo-EM	5.31e-3	0.095	3.22e-5	4.50e-6	1.68e-8	0.246	0.135	0.378	0.427	0.527
6peo [29]	α-syn	human recombinant	Cryo-EM	1.49e-6	1.05e-4	7.81e-12	6.60e-8	1.11e-9	0.395	0.333	0.588	0.470	0.526
6pes [29]	α-syn	recombinant	Cryo-EM	9.40e-7<br>3.31e-7	5.79e-5<br>5.97e-4	2.46e-12<br>5.49e-11	2.80e-8<br>2.57e-7	5.00e-10<br>1.10e-9	0.407<br>0.467	0.346<br>0.287	0.602<br>0.557	0.483<br>0.455	0.540<br>0.549
6rt0 [30]	α-syn	human, recombinant	Cryo-EM	9.56e-10	5.02e-10	8.38e-15	1.15e-7	7.53e-11	0.461	0.529	0.652	0.448	0.537

6rtb [30]	α-syn	human, recombinant	Cryo-EM	9.15e-9	2.23e-13	7.72e-21	6.12e-9	4.12e-12	0.471	0.626	0.786	0.504	0.596
6sst [30]	α-syn	human, recombinant	Cryo-EM	1.17e-8	3.52e-10	1.18e-16	3.30e-7	1.70e-10	0.408	0.526	0.677	0.420	0.508
6ssx [30]	α-syn	human, recombinant	Cryo-EM	1.17e-8	3.52e-10	1.18e-16	3.30e-7	1.70e-10	0.408	0.526	0.677	0.420	0.508
6ufr [31]	α-syn	recombinant	Cryo-EM	2.09e-6	1.53e-4	2.99e-9	4.75e-10	1.72e-12	0.367	0.322	0.507	0.526	0.583
6xyo [32]	α-syn	human, extracted	Cryo-EM	2.17e-3<br>2.16e-4	0.055<br>1.72e-5	3.33e-6<br>2.54e-13	7.18e-7<br>4.06e-6	9.07e-9<br>5.69e-8	0.227<br>0.268	0.150<br>0.361	0.393<br>0.602	0.412<br>0.377	0.468<br>0.433
6xyp [32]	α-syn	human, extracted	Cryo-EM	1.20e-4<br>7.29e-3	2.85e-3<br>8.61e-4	2.86e-10<br>4.94e-8	1.27e-5<br>1.76e-4	7.42e-14<br>1.21e-3	0.324<br>0.208	0.252<br>0.283	0.545<br>0.469	0.383<br>0.320	0.655<br>0.271
6xyq [32]	α-syn	human, extracted	Cryo-EM	4.93e-7<br>1.58e-4	1.46e-4<br>4.70e-5	7.96e-12<br>1.79e-12	1.82e-9<br>2.33e-6	1.78e-12<br>3.14e-12	0.441<br>0.276	0.324<br>0.343	0.588<br>0.585	0.531<br>0.389	0.626<br>0.444
7c1d [33]	α-syn	recombinant, seeded	Cryo-EM	2.48e-3	0.013	1.07e-6	2.58e-3	2.38e-8	0.277	0.167	0.340	0.226	0.452
7e0f [34]	α-syn	recombinant	Cryo-EM	1.13e-6	1.49e-4	5.75e-11	4.14e-7	2.93e-8	0.451	0.314	0.551	0.445	0.500
7l7h [35]	α-syn	recombinant	Cryo-EM	0.195<br>0.412	0.310<br>0.459	9.68e-3<br>0.200	0.342<br>0.292	2.38e-8<br>7.88e-6	0.096<br>0.043	0.050<br>-0.028	0.180<br>0.060	0.049<br>-0.058	0.452<br>0.308
7nca [31]	α-syn	human, recombinant,<br>seeded	Cryo-EM	5.85e-5<br>5.79e-6	2.21e-3<br>1.04e-4	2.49e-5<br>1.92e-10	1.97e-7<br>2.94e-8	2.40e-3<br>1.06e-10	0.386<br>0.431	0.225<br>0.312	0.319<br>0.518	0.425<br>0.481	0.250<br>0.579
7ncg [36]	α-syn	human recombinant<br>seeded	Cryo-EM	1.24e-8	6.81e-6	2.00e-11	3.37e-11	1.76e-15	0.437	0.385	0.570	0.558	0.650
7nch [36]	α-syn	recombinant seeded	Cryo-EM	0.060<br>0.060	0.202<br>0.010	5.79e-3<br>2.49e-5	0.072<br>0.072	1.29e-2<br>1.29e-2	0.163<br>0.163	0.074<br>0.187	0.203<br>0.319	0.127<br>0.127	0.205<br>0.205
7nci [36]	α-syn	human recombinant<br>seeded	Cryo-EM	0.019	0.069	5.56e-6	0.290	1.29e-5	0.215	0.105	0.279	0.056	0.325
7ncj [36]	α-syn	human recombinant<br>seeded	Cryo-EM	0.019<br>1.37e-6	0.034<br>9.14e-5	1.50e-6<br>1.25e-9	0.131<br>2.11e-10	2.84e-6<br>6.55e-16	0.199<br>0.377	0.131<br>0.334	0.305<br>0.520	0.088<br>0.539	0.358<br>0.664
7nck [36]	α-syn	human recombinant<br>seeded	Cryo-EM	8.46e-6	2.58e-4	3.94e-11	2.80e-8	6.35e-9	0.369	0.313	0.569	0.483	0.504

7ozg [37]	α-syn	recombinant, seeded	Cryo-EM	7.26e-9	1.44e-10	3.58e-17	1.79e-7	4.18e-12	0.418	0.539	0.691	0.432	0.552
7ozh [37]	α-syn	recombinant seeded	Cryo-EM	4.50e-10	5.99e-7	7.81e-12	5.25e-10	1.43e-16	0.509	0.430	0.588	0.538	0.702
7uak [38]	α-syn	human recombinant	Cryo-EM	5.82e-6	1.07e-3	3.67e-7	5.11e-6	1.90e-10	0.409	0.274	0.436	0.403	0.573
7v47 [39]	α-syn	human recombinant seeded	Cryo-EM	0.020	3.74e-3	1.86e-7	1.16e-3	3.60e-13	0.198	0.235	0.436	0.280	0.653
7v48 [39]	α-syn	human recombinant seeded	Cryo-EM	9.85e-4	5.97e-4	1.25e-7	1.70e-5	6.49e-11	0.289	0.287	0.451	0.379	0.588
7v49 [39]	α-syn	human recombinant seeded	Cryo-EM	6.27e-4	1.24e-4	5.96e-6	4.60e-10	2.08e-3	0.321	0.268	0.325	0.474	0.247
7v4a [40]	α-syn	human recombinant	Cryo-EM	1.86e-8	5.13e-7	4.64e-12	7.34e-8	3.58e-10	0.398	0.422	0.572	0.440	0.496
7v4b [40]	α-syn	human recombinant	Cryo-EM	2.71e-9	2.98e-9	3.35e-15	4.95e-8	1.91e-11	0.439	0.503	0.656	0.456	0.545
7v4c [40]	α-syn	human recombinant	Cryo-EM	6.33e-9	1.81e-6	3.34e-9	8.76e-11	1.97e-3	0.565	0.377	0.474	0.552	0.269
7v4d [41]	α-syn	human recombinant	Cryo-EM	0.056	0.241	4.24e-5	0.031	2.46e-7	0.162	0.069	0.341	0.176	0.464
7wmm [42]	α-syn	human recombinant	Cryo-EM	1.37e-6	4.75e-4	1.23e-9	6.66e-11	3.32e-9	0.416	0.298	0.525	0.573	0.523
7wnz [43]	α-syn	human recombinant	Cryo-EM	1.45e-4	2.52e-3	3.75e-9	1.06e-6	2.66e-8	0.309	0.255	0.509	0.423	0.478
7wo0 [44]	α-syn	human recombinant	Cryo-EM	8.48e-11	2.13e-7	7.96e-14	1.46e-12	8.60e-18	0.473	0.441	0.625	0.584	0.675
7xjx [45]	α-syn	human recombinant	Cryo-EM	6.27e-3	0.272	4.79e-4	3.14e-5	3.58e-8	0.234	0.066	0.296	0.366	0.495
7xo0 [39]	α-syn	human recombinant seeded	Cryo-EM	3.57e-7	3.86e-7	2.47e-13	2.26e-10	3.84e-10	0.410	0.437	0.626	0.545	0.534
7xo1 [39]	α-syn	human recombinant seeded	Cryo-EM	1.28e-5	3.90e-6	4.40e-13	4.99e-7	1.77e-13	0.357	0.398	0.621	0.436	0.632
7xo2 [39]	α-syn	human recombinant seeded	Cryo-EM	6.94e-5	1.12e-5	2.46e-12	2.04e-6	1.62e-12	0.330	0.378	0.602	0.414	0.611

7xo3 [39]	α-syn	human recombinant seeded	Cryo-EM	6.97e-4	7.65e-4	2.74e-7	1.84e-5	8.63e-11	0.298	0.282	0.443	0.376	0.581
7yk2 [46]	α-syn	human recombinant	Cryo-EM	1.62e-3	0.112	3.10e-5	1.53e-6	7.49e-10	0.280	0.115	0.350	0.421	0.556
7yk8 [46]	α-syn	human recombinant	Cryo-EM	3.93e-4	3.37e-3	5.88e-8	1.29e-5	2.91e-5	0.292	0.245	0.470	0.380	0.363
7yng [47]	α-syn	human recombinant	Cryo-EM	4.50e-5	1.07e-3	2.13e-10	6.42e-8	1.90e-10	0.365	0.274	0.542	0.478	0.573
7ynl [48]	α-syn	human recombinant	Cryo-EM	0.018	0.050	1.97e-7	6.77e-5	7.35e-7	0.193	0.155	0.450	0.350	0.439
7ynm [49]	α-syn	human recombinant	Cryo-EM	2.37e-3	0.017	9.14e-7	1.53e-7	5.23e-10	0.266	0.192	0.423	0.464	0.558
7ynn [50]	α-syn	human recombinant	Cryo-EM	7.61e-3	0.029	4.31e-7	4.72e-7	1.73e-8	0.224	0.176	0.437	0.445	0.500
7yno [51]	α-syn	human recombinant	Cryo-EM	5.16e-3	7.90e-3	1.22e-6	2.54e-8	1.10e-9	0.245	0.218	0.415	0.492	0.549
7ynp [52]	α-syn	human recombinant	Cryo-EM	2.57e-3	0.016	1.32e-7	1.07e-7	3.32e-9	0.254	0.198	0.457	0.467	0.523
7ynq [53]	α-syn	human recombinant	Cryo-EM	5.16e-3	7.90e-3	1.22e-6	2.54e-8	1.10e-9	0.245	0.218	0.415	0.492	0.549
7ynr [54]	α-syn	human recombinant	Cryo-EM	5.51e-6	6.02e-4	1.85e-10	8.46e-11	3.29e-8	0.380	0.293	0.550	0.566	0.482
7yns [55]	α-syn	human recombinant	Cryo-EM	5.51e-6	6.02e-4	1.85e-10	8.46e-11	3.29e-8	0.380	0.293	0.550	0.566	0.482
7ynt [56]	α-syn	human recombinant	Cryo-EM	1.70e-3	0.076	2.84e-6	1.80e-4	3.59e-7	0.278	0.136	0.401	0.329	0.456
8a4l [57]	α-syn	human recombinant	Cryo-EM	1.07e-6	4.28e-7	4.33e-12	5.00e-6	9.07e-9	0.361	0.429	0.580	0.380	0.468
8a9l [58]	α-syn	human, extracted	Cryo-EM	7.97e-7	5.93e-7	1.14e-13	1.11e-10	1.66e-11	0.317	0.403	0.577	0.487	0.485
8ads [57]	α-syn	human, recombinant	Cryo-EM	1.30e-7	1.25e-7	6.69e-13	1.34e-6	1.38e-9	0.385	0.447	0.598	0.400	0.488
8adu [57]	α-syn	human, recombinant	Cryo-EM	5.55e-6	1.45e-7	6.71e-13	3.79e-5	9.81e-8	0.324	0.440	0.589	0.335	0.422

8adv [57]	α-syn	human, recombinant	Cryo-EM	5.54e-7	3.06e-11	4.58e-14	1.74e-4	3.57e-9	0.325	0.532	0.590	0.292	0.437
8adw [57]	α-syn	human, recombinant	Cryo-EM	9.84e-6	8.92e-10	2.56e-12	5.62e-5	8.03e-8	0.288	0.490	0.548	0.311	0.397
8aex [57]	α-syn	human, recombinant	Cryo-EM	2.21e-7<br>3.59e-6	1.29e-6<br>5.22e-8	1.44e-12<br>4.46e-12	1.23e-9<br>6.64e-5	8.03e-8<br>1.73e-10	0.421<br>0.373	0.417<br>0.469	0.607<br>0.593	0.525<br>0.343	0.548<br>0.397
8cyr [59]	α-syn	human, recombinant	Cryo-EM	7.04e-7	2.13e-7	1.60e-12	4.11e-7	4.73e-9	0.371	0.441	0.593	0.424	0.480
8cys [59]	α-syn	human, recombinant, seeded	Cryo-EM	7.78e-4	4.09e-5	3.38e-10	2.83e-3	4.66e-13	0.272	0.353	0.542	0.253	0.617
8cyt [59]	α-syn	human, recombinant, seeded	Cryo-EM	4.63e-7	1.45e-7	3.16e-14	1.36e-7	9.44e-9	0.356	0.440	0.620	0.428	0.453
8cyv [59]	α-syn	human, recombinant, seeded	Cryo-EM	6.73e-5<br>2.01e-7	1.01e-5<br>3.35e-8	4.99e-10<br>3.66e-15	9.46e-7<br>3.87e-8	6.12e-5<br>3.14e-8	0.316<br>0.375	0.379<br>0.465	0.533<br>0.646	0.419<br>0.451	0.339<br>0.444
8cyw [59]	α-syn	human, recombinant, seeded	Cryo-EM	1.24e-8	2.05e-8	6.08e-14	3.32e-6	5.50e-10	0.437	0.480	0.634	0.395	0.517
8cyx [59]	α-syn	human, recombinant, seeded	Cryo-EM	5.67e-7	1.19e-7	1.31e-11	2.17e-5	8.55e-7	0.399	0.456	0.579	0.365	0.419
8cyy [59]	α-syn	human, recombinant, seeded	Cryo-EM	2.15e-5<br>6.99e-8	1.69e-6<br>7.88e-8	1.79e-12<br>9.55e-15	3.77e-9<br>5.11e-9	2.85e-7<br>2.52e-9	0.309<br>0.369	0.404<br>0.445	0.585<br>0.624	0.483<br>0.466	0.413<br>0.462
8cz0 [59]	α-syn	human, recombinant, seeded	Cryo-EM	1.45e-4	1.29e-6	1.44e-12	1.90e-4	1.66e-14	0.309	0.417	0.607	0.321	0.652
8cz1 [59]	α-syn	human, recombinant, seeded	Cryo-EM	3.06e-7	6.99e-8	1.08e-14	7.34e-8	4.97e-9	0.365	0.452	0.633	0.440	0.465
8cz2 [59]	α-syn	human, recombinant, seeded	Cryo-EM	4.54e-8	3.83e-8	3.29e-15	2.80e-8	1.34e-9	0.379	0.458	0.637	0.447	0.473
8cz3 [59]	α-syn	human, recombinant, seeded	Cryo-EM	3.06e-7	6.99e-8	1.08e-14	7.34e-8	4.97e-9	0.365	0.452	0.633	0.440	0.465
8cz6 [59]	α-syn	human, recombinant, seeded	Cryo-EM	2.58e-6	2.57e-7	8.83e-14	3.30e-7	3.14e-8	0.342	0.434	0.615	0.420	0.444
8fpt [60]	α-syn	human, recombinant, seeded	ss-NMR	8.07e-8	5.79e-5	3.94e-11	2.15e-10	4.32e-6	0.445	0.346	0.569	0.552	0.398

8h03 [39]	α-syn	human recombinant seeded	Cryo-EM	6.97e-4	7.65e-4	2.74e-7	1.84e-5	8.63e-11	0.298	0.282	0.443	0.376	0.581
8h04 [39]	α-syn	human recombinant seeded	Cryo-EM	6.97e-4	7.65e-4	2.74e-7	1.84e-5	8.63e-11	0.298	0.282	0.443	0.376	0.581
8h05 [39]	α-syn	human recombinant seeded	Cryo-EM	6.92e-4	1.36e-3	4.27e-7	9.66e-7	4.31e-5	0.301	0.266	0.429	0.431	0.366

Table S4. Amyloid fibril/protofilament structures of αS considering L ≥ 1 deposited in the PDB and listed in the *Amyloid Atlas*.[1]

PDB ID	Protein	Fibril origin	Method	*p* (one-tail Fisher's exact test)					*κ* (Cohen's kappa coefficient test)				
				ZYGGR.	AGGRES.	TANGO	WALTZ	PASTA2.0	ZYGGR.	AGGRES.	TANGO	WALTZ	PASTA2.0
2n0a [22]	α-syn	human recombinant	ss-NMR	4.74e-8	1.46e-4	2.39e-8	1.14e-9	2.18e-10	0.457	0.326	0.483	0.531	0.554
6a6b [23]	α-syn	human recombinant	Cryo-EM	1.01e-4	0.014	6.30e-6	6.60e-8	1.11e-9	0.320	0.203	0.391	0.470	0.526
6cu7 [23]	α-syn	human recombinant	Cryo-EM	1.01e-4	0.035	3.20e-5	3.81e-6	1.11e-9	0.320	0.170	0.358	0.402	0.526
6cu8 [23]	α-syn	human recombinant	Cryo-EM	8.59e-3	0.112	3.10e-5	4.40e-4	1.02e-15	0.234	0.115	0.350	0.306	0.717
6h6b [24]	α-syn	human recombinant	Cryo-EM	2.02e-5	6.61e-3	6.70e-6	3.39e-7	4.79e-9	0.367	0.243	0.414	0.469	0.533
6l1t [25]	α-syn	semi-synthetic	Cryo-EM	5.15e-3	1.96e-5	1.92e-7	3.17e-4	1.65e-6	0.153	0.310	0.367	0.243	0.303
6l1u [25]	α-syn	semi-synthetic	Cryo-EM	0.029<br>0.013<br>0.016	7.31e-5<br>3.90e-5<br>6.30e-5	9.00e-6<br>3.99e-6<br>6.69e-6	4.55e-3<br>6.70e-4<br>9.51e-4	2.63e-7<br>6.71e-7<br>1.06e-6	0.123<br>0.138<br>0.132	0.298<br>0.304<br>0.294	0.327<br>0.334<br>0.324	0.198<br>0.234<br>0.225	0.338<br>0.320<br>0.311
6l4s [26]	α-syn	recombinant	Cryo-EM	3.53e-6	1.32e-3	9.81e-9	5.28e-7	7.35e-15	0.391	0.272	0.497	0.440	0.676
6lrq [27]	α-syn	recombinant	Cryo-EM	7.31e-8	2.26e-4	5.41e-9	9.07e-11	2.98e-13	0.424	0.315	0.501	0.552	0.610
6osj [28]	α-syn	human recombinant	Cryo-EM	3.59e-6	0.011	4.06e-6	3.14e-8	3.84e-10	0.373	0.211	0.398	0.478	0.534
6osl [28]	α-syn	human recombinant	Cryo-EM	1.21e-5	0.011	2.73e-6	7.47e-8	4.99e-10	0.360	0.224	0.427	0.485	0.549
6peo [29]	α-syn	human recombinant	Cryo-EM	2.96e-7	6.23e-5	5.31e-11	1.12e-9	3.94e-12	0.391	0.341	0.557	0.513	0.570
6pes [29]	α-syn	recombinant	Cryo-EM	1.88e-7<br>8.07e-8	3.51e-5<br>9.98e-4	2.00e-11<br>5.88e-8	4.75e-10<br>2.80e-8	1.72e-12<br>3.18e-11	0.402<br>0.445	0.354<br>0.280	0.570<br>0.470	0.526<br>0.483	0.583<br>0.575
6rt0 [30]	α-syn	human, recombinant	Cryo-EM	4.46e-9	6.90e-9	2.30e-13	9.52e-8	4.05e-11	0.428	0.490	0.611	0.444	0.532

6rtb [30]	α-syn	human, recombinant	Cryo-EM	1.18e-7	7.15e-11	2.16e-17	2.76e-8	1.56e-11	0.413	0.556	0.712	0.473	0.563
6sst [30]	α-syn	human, recombinant	Cryo-EM	2.92e-9	1.92e-9	1.13e-15	1.36e-7	3.81e-11	0.388	0.500	0.650	0.428	0.516
6ssx [30]	α-syn	human, recombinant	Cryo-EM	4.54e-8	4.29e-9	3.29e-15	2.80e-8	7.64e-11	0.379	0.488	0.637	0.447	0.504
6ufr [31]	α-syn	recombinant	Cryo-EM	2.09e-6	1.53e-4	2.99e-9	4.75e-10	1.72e-12	0.367	0.322	0.507	0.526	0.583
6xyo [32]	α-syn	human, extracted	Cryo-EM	4.43e-5<br>9.84e-6	0.015<br>3.72e-6	6.05e-6<br>4.42e-11	6.00e-9<br>2.01e-7	2.00e-9<br>6.51e-9	0.273<br>0.288	0.192<br>0.373	0.366<br>0.519	0.451<br>0.399	0.448<br>0.426
6xyp [32]	α-syn	human, extracted	Cryo-EM	5.67e-7<br>1.21e-4	1.32e-4<br>3.25e-5	1.93e-10<br>1.49e-10	6.16e-8<br>3.00e-5	3.64e-15<br>4.66e-9	0.399<br>0.267	0.327<br>0.344	0.547<br>0.521	0.465<br>0.333	0.658<br>0.450
6xyq [32]	α-syn	human, extracted	Cryo-EM	7.42e-9<br>1.12e-4	9.14e-5<br>1.51e-4	1.25e-9<br>1.36e-8	1.40e-11<br>3.50e-7	2.56e-14<br>1.15e-8	0.448<br>0.250	0.334<br>0.304	0.520<br>0.449	0.572<br>0.388	0.630<br>0.416
7c1d [33]	α-syn	recombinant, seeded	Cryo-EM	1.34e-4	2.15e-3	4.30e-9	2.14e-8	1.33e-9	0.353	0.248	0.488	0.490	0.546
7e0f [34]	α-syn	recombinant	Cryo-EM	2.15e-8	7.20e-5	3.82e-11	6.82e-10	3.85e-8	0.496	0.337	0.568	0.545	0.491
7l7h [35]	α-syn	recombinant	Cryo-EM	0.017<br>5.28e-4	0.016<br>6.98e-3	7.07e-5<br>2.20e-5	0.011<br>3.31e-3	3.48e-12<br>6.94e-13	0.205<br>0.324	0.174<br>0.207	0.305<br>0.338	0.199<br>0.241	0.596<br>0.630
7nca [31]	α-syn	human, recombinant, seeded	Cryo-EM	1.77e-6<br>2.85e-5	6.50e-6<br>5.97e-4	2.66e-8<br>1.25e-7	3.73e-8<br>2.13e-6	8.63e-11<br>6.49e-11	0.426<br>0.378	0.385<br>0.287	0.478<br>0.451	0.486<br>0.417	0.581<br>0.588
7ncg [36]	α-syn	human recombinant seeded	Cryo-EM	8.33e-9	7.23e-5	7.54e-10	3.49e-12	6.63e-14	0.395	0.336	0.518	0.571	0.597
7nch [36]	α-syn	recombinant seeded	Cryo-EM	2.39e-5<br>5.54e-5	7.20e-5<br>1.06e-5	6.44e-7<br>5.52e-8	3.75e-5<br>1.18e-4	2.32e-10<br>1.43e-9	0.371<br>0.347	0.337<br>0.378	0.430<br>0.471	0.363<br>0.337	0.566<br>0.537
7nci [36]	α-syn	human recombinant seeded	Cryo-EM	1.07e-5	1.49e-4	1.39e-6	2.71e-5	5.94e-12	0.405	0.314	0.408	0.369	0.620
7ncj [36]	α-syn	human recombinant seeded	Cryo-EM	5.82e-6<br>2.03e-8	8.32e-6<br>1.28e-5	3.67e-7<br>5.31e-11	3.14e-5<br>4.77e-12	1.90e-10<br>4.57e-15	0.409<br>0.426	0.378<br>0.373	0.436<br>0.557	0.366<br>0.578	0.573<br>0.636
7nck [36]	α-syn	human recombinant seeded	Cryo-EM	2.96e-7	6.23e-5	6.61e-10	7.82e-11	3.94e-12	0.391	0.341	0.525	0.545	0.570
7ozg [37]	α-syn	recombinant, seeded	Cryo-EM	2.92e-8	1.92e-9	1.13e-15	1.36e-7	1.63e-12	0.388	0.500	0.650	0.428	0.547

7ozh [37]	α-syn	recombinant seeded	Cryo-EM	8.30e-12	7.25e-8	3.98e-13	7.51e-13	6.55e-16	0.520	0.461	0.616	0.605	0.664
7uak [38]	α-syn	human recombinant	Cryo-EM	8.31e-7	1.03e-3	4.31e-7	4.82e-8	9.57e-11	0.428	0.277	0.437	0.481	0.574
7v47 [39]	α-syn	human recombinant seeded	Cryo-EM	0.012	3.00e-3	2.74e-7	1.84e-5	8.63e-11	0.213	0.248	0.443	0.376	0.581
7v48 [39]	α-syn	human recombinant seeded	Cryo-EM	7.63e-6	5.76e-5	5.00e-9	4.82e-8	2.19e-13	0.387	0.344	0.505	0.481	0.648
7v49 [39]	α-syn	human recombinant seeded	Cryo-EM	6.93e-5	1.83e-3	3.76e-5	1.51e-8	7.24e-9	0.353	0.261	0.353	0.501	0.519
7v4a [40]	α-syn	human recombinant	Cryo-EM	3.76e-7	3.38e-5	1.29e-10	6.32e-8	2.00e-9	0.334	0.339	0.514	0.421	0.448
7v4b [40]	α-syn	human recombinant	Cryo-EM	3.76e-7	6.62e-6	8.49e-12	3.84e-6	2.00e-9	0.334	0.368	0.543	0.361	0.448
7v4c [40]	α-syn	human recombinant	Cryo-EM	4.60e-9	1.06e-5	4.16e-10	3.62e-10	1.73e-7	0.510	0.378	0.539	0.552	0.463
7v4d [41]	α-syn	human recombinant	Cryo-EM	3.53e-6	1.32e-3	8.44e-8	5.28e-7	7.35e-15	0.391	0.272	0.463	0.440	0.676
7wmm [42]	α-syn	human recombinant	Cryo-EM	1.88e-7	1.53e-4	2.99e-9	3.37e-11	3.48e-11	0.402	0.322	0.507	0.558	0.550
7wnz [43]	α-syn	human recombinant	Cryo-EM	7.64e-6	6.96e-4	3.35e-8	4.69e-9	1.91e-11	0.337	0.286	0.468	0.488	0.545
7wo0 [44]	α-syn	human recombinant	Cryo-EM	2.48e-10	8.71e-7	6.69e-13	8.13e-12	5.94e-17	0.451	0.416	0.598	0.558	0.648
7xjx [45]	α-syn	human recombinant	Cryo-EM	7.53e-4	0.113	1.29e-3	1.27e-5	3.66e-11	0.284	0.119	0.275	0.383	0.582
7xo0 [39]	α-syn	human recombinant seeded	Cryo-EM	8.88e-7	1.77e-6	2.38e-12	9.07e-11	1.22e-10	0.388	0.411	0.598	0.552	0.542
7xo1 [39]	α-syn	human recombinant seeded	Cryo-EM	1.37e-6	3.53e-6	6.84e-12	2.82e-9	7.28e-13	0.377	0.398	0.584	0.506	0.597
7xo2 [39]	α-syn	human recombinant seeded	Cryo-EM	8.15e-6	1.01e-5	3.64e-11	1.41e-8	6.83e-12	0.352	0.379	0.566	0.486	0.576
7xo3 [39]	α-syn	Human recombinant seeded	Cryo-EM	9.40e-7	1.12e-5	5.20e-10	2.70e-9	1.62e-12	0.407	0.378	0.536	0.518	0.611

7yk2 [46]	α-syn	human recombinant	Cryo-EM	5.51e-6	2.15e-3	1.53e-6	1.28e-8	2.18e-10	0.380	0.260	0.417	0.497	0.554
7yk8 [46]	α-syn	human recombinant	Cryo-EM	1.58e-4	7.65e-4	3.69e-8	2.33e-6	2.85e-7	0.276	0.281	0.462	0.389	0.413
7yng [47]	α-syn	human recombinant	Cryo-EM	5.45e-6	1.87e-3	1.82e-7	6.64e-9	5.48e-11	0.362	0.263	0.450	0.498	0.556
7ynl [48]	α-syn	human recombinant	Cryo-EM	5.45e-6	5.22e-4	2.19e-8	6.64e-9	8.25e-10	0.362	0.295	0.482	0.498	0.521
7ynm [49]	α-syn	human recombinant	Cryo-EM	8.15e-6	8.61e-4	4.94e-8	1.19e-9	1.22e-10	0.352	0.283	0.469	0.519	0.542
7ynn [50]	α-syn	human recombinant	Cryo-EM	1.35e-5	1.37e-3	1.07e-7	2.82e-9	2.62e-10	0.342	0.271	0.456	0.506	0.529
7yno [51]	α-syn	human recombinant	Cryo-EM	5.45e-6	1.87e-3	1.82e-7	6.16e-8	2.89e-12	0.362	0.263	0.450	0.465	0.590
7ynp [52]	α-syn	human recombinant	Cryo-EM	2.09e-6	6.17e-4	2.82e-8	4.75e-10	3.48e-11	0.367	0.291	0.475	0.526	0.550
7ynq [53]	α-syn	human recombinant	Cryo-EM	5.45e-6	1.87e-3	1.82e-7	6.16e-8	2.89e-12	0.362	0.263	0.450	0.465	0.590
7ynr [54]	α-syn	human recombinant	Cryo-EM	1.88e-7	1.53e-4	2.99e-9	3.37e-11	3.48e-11	0.402	0.322	0.507	0.558	0.550
7yns [55]	α-syn	human recombinant	Cryo-EM	1.88e-7	1.53e-4	2.99e-9	3.37e-11	3.48e-11	0.402	0.322	0.507	0.558	0.550
7ynt [56]	α-syn	human recombinant	Cryo-EM	3.20e-5	3.86e-3	6.28e-7	3.14e-8	4.95e-9	0.336	0.243	0.430	0.478	0.500
8a4l [57]	α-syn	human recombinant	Cryo-EM	1.63e-5	6.39e-6	1.06e-11	7.81e-7	5.50e-8	0.298	0.374	0.551	0.394	0.420
8a9l [58]	α-syn	human, extracted	Cryo-EM	1.18e-6	1.10e-6	2.80e-13	2.19e-10	3.17e-11	0.309	0.391	0.565	0.476	0.474
8ads [57]	α-syn	human, recombinant	Cryo-EM	2.31e-6	2.02e-6	1.48e-12	1.74e-7	8.48e-9	0.320	0.391	0.568	0.413	0.439
8adu [57]	α-syn	human, recombinant	Cryo-EM	4.65e-4	1.84e-5	3.91e-8	6.20e-4	1.20e-5	0.215	0.335	0.420	0.252	0.310
8adv [57]	α-syn	human, recombinant	Cryo-EM	3.36e-5	1.94e-8	6.76e-10	9.36e-4	1.63e-7	0.236	0.419	0.448	0.234	0.347
8adw [57]	α-syn	human, recombinant	Cryo-EM	2.82e-4	1.35e-6	9.02e-9	2.21e-4	1.75e-6	0.209	0.364	0.421	0.261	0.320

8aex [57]	α-syn	human, recombinant	Cryo-EM	1.37e-6<br>2.88e-5	1.93e-5<br>3.50e-7	1.04e-10<br>1.86e-13	2.82e-9<br>8.93e-7	3.51e-9<br>1.37e-7	0.377<br>0.322	0.366<br>0.435	0.552<br>0.620	0.506<br>0.415	0.496<br>0.438
8cyr [59]	α-syn	human, recombinant	Cryo-EM	5.55e-6	9.98e-7	6.71e-13	1.36e-7	9.44e-9	0.324	0.410	0.589	0.428	0.453
8cys [59]	α-syn	human, recombinant, seeded	Cryo-EM	2.47e-3	3.78e-4	1.22e-8	2.78e-4	7.28e-13	0.235	0.303	0.488	0.308	0.597
8cyt [59]	α-syn	human, recombinant, seeded	Cryo-EM	4.63e-7	1.45e-7	3.16e-14	1.36e-7	9.44e-9	0.356	0.440	0.620	0.428	0.453
8cyv [59]	α-syn	human, recombinant, seeded	Cryo-EM	4.60e-5<br>2.01e-7	5.90e-6<br>3.35e-8	1.16e-11<br>3.66e-15	1.47e-8<br>3.87e-8	9.81e-8<br>3.14e-8	0.292<br>0.375	0.379<br>0.465	0.559<br>0.646	0.459<br>0.451	0.422<br>0.444
8cyw [59]	α-syn	human, recombinant, seeded	Cryo-EM	3.29e-8	1.01e-7	5.43e-13	1.57e-6	1.59e-10	0.416	0.454	0.607	0.404	0.525
8cyx [59]	α-syn	human, recombinant, seeded	Cryo-EM	1.30e-7	1.54e-8	3.09e-14	1.98e-8	8.40e-11	0.385	0.477	0.629	0.463	0.520
8cyy [59]	α-syn	human, recombinant, seeded	Cryo-EM	4.60e-5<br>6.99e-8	5.90e-6<br>7.88e-8	1.16e-11<br>9.55e-15	1.30e-9<br>5.11e-9	9.81e-8<br>2.52e-9	0.292<br>0.369	0.379<br>0.445	0.559<br>0.624	0.490<br>0.466	0.422<br>0.462
8cz0 [59]	α-syn	human, recombinant, seeded	Cryo-EM	5.41e-4	1.93e-5	1.04e-10	1.15e-5	2.56e-14	0.270	0.366	0.552	0.374	0.630
8cz1 [59]	α-syn	human, recombinant, seeded	Cryo-EM	7.40e-7	2.91e-7	8.66e-14	2.80e-8	1.34e-9	0.347	0.428	0.606	0.447	0.473
8cz2 [59]	α-syn	human, recombinant, seeded	Cryo-EM	6.99e-8	7.88e-8	9.55e-15	5.25e-8	2.52e-9	0.369	0.445	0.624	0.436	0.462
8cz3 [59]	α-syn	human, recombinant, seeded	Cryo-EM	3.06e-7	6.99e-8	1.08e-14	7.34e-8	4.97e-9	0.365	0.452	0.633	0.440	0.465
8cz6 [59]	α-syn	human, recombinant, seeded	Cryo-EM	2.58e-6	2.57e-7	8.83e-14	3.30e-7	3.14e-8	0.342	0.434	0.615	0.420	0.444
8fpt [60]	α-syn	human, recombinant, seeded	ss-NMR	5.17e-6	1.53e-3	1.55e-8	1.76e-10	1.91e-6	0.347	0.267	0.481	0.532	0.393
8h03 [39]	α-syn	human recombinant seeded	Cryo-EM	9.40e-7	1.12e-5	5.20e-10	2.70e-9	1.62e-12	0.407	0.378	0.536	0.518	0.611
8h04 [39]	α-syn	human recombinant seeded	Cryo-EM	3.53e-6	8.03e-5	9.81e-9	5.59e-8	2.18e-13	0.391	0.339	0.497	0.475	0.640

8h05 [39]	α-syn	human recombinant seeded	Cryo-EM	3.53e-6	8.03e-5	7.15e-7	5.60e-9	9.11e-11	0.391	0.339	0.430	0.510	0.568

Table S5. Amyloid fibril/protofilament structures of 4R tau considering L ≥ 4 deposited in the PDB and listed in the *Amyloid Atlas*.[1]

PDB ID	Protein	Fibril origin	Method	*p* (one-tail Fisher's exact test)					*κ* (Cohen's kappa coefficient test)				
				ZYGGR.	AGGRES.	TANGO	WALTZ	PASTA2.0	ZYGGR.	AGGRES.	TANGO	WALTZ	PASTA2.0
8cax[61]	2N4R tau	human, extracted	Cryo-EM	1.00e-4	1.00e-3	3.20e-3	1.10e-3	1.07e-2	0.269	0.161	0.138	0.175	0.121
6tjo[62]	2N4R tau	human, extracted	Cryo-EM	1.00e-4	2.14e-2	9.60e-3	1.00e-3	0.108	0.263	9.30e-2	0.103	0.170	5.97e-2
7p6d[63]	2N4R tau	human, extracted	Cryo-EM	1.00e-4	9.00e-4	9.40e-3	1.00e-4	2.00e-4	0.215	0.116	0.083	0.339	0.133
8orf[64]	1N4R tau	human, seeded	Cryo-EM	1.00e-4	1.00e-3	2.30e-3	1.00e-4	1.80e-3	0.263	0.135	0.118	0.293	0.129
8org[64]	1N4R tau	human, seeded	Cryo-EM	1.00e-4	1.00e-3	2.30e-3	1.00e-4	1.80e-3	0.263	0.135	0.118	0.293	0.129
6tjx[62]	2N4R tau	human, extracted	Cryo-EM	1.00e-4	5.80e-2	2.78e-2	1.00e-4	8.00e-4	0.284	6.39e-2	7.32e-2	0.247	0.129
7p6e[63]	2N4R tau	human, extracted	Cryo-EM	1.00e-4	6.00e-4	1.50e-3	1.00e-4	1.00e-4	0.383	0.148	0.130	0.316	0.199
7p65[63]	2N4R tau	human, extracted	Cryo-EM	1.00e-4	4.98e-2	9.49e-2	1.00e-4	6.00e-4	0.229	6.82e-2	5.30e-2	0.336	0.136
7u0z[65]	2N4R tau	human, extracted	Cryo-EM	1.00e-4	4.98e-2	9.49e-2	1.00e-4	6.00e-4	0.229	6.82e-2	5.30e-2	0.355	0.136
7p66[63]	2N4R tau	human, extracted	Cryo-EM	1.00e-4	1.03e-2	3.60e-3	1.00e-4	1.00e-4	0.309	9.60e-2	0.106	0.326	0.165
7p67[63]	2N4R tau	human, extracted	Cryo-EM	1.00e-4	7.70e-3	2.70e-3	1.00e-4	1.00e-4	0.327	0.104	0.114	0.264	0.176
7p68[63]	2N4R tau	human, extracted	Cryo-EM	1.00e-4<br>1.00e-4	7.10e-3<br>3.16e-2	2.50e-3<br>1.45e-2	1.00e-4<br>1.00e-4	1.00e-4<br>1.00e-4	0.308<br>0.337	0.106<br>0.081	0.116<br>0.091	0.268<br>0.231	0.179<br>0.182
7p6a[63]	2N4R tau	human, extracted	Cryo-EM	1.00e-4	1.90e-3	3.90e-3	1.00e-4	1.00e-4	0.305	0.119	0.104	0.321	0.162
7p6b[63]	2N4R tau	human, extracted	Cryo-EM	1.00e-4	1.80e-3	3.60e-3	1.00e-4	1.00e-4	0.309	0.121	0.106	0.326	0.165

7p6c[63]	2N4R tau	human, extracted	Cryo-EM	1.00e-4	1.80e-3	3.60e-3	1.00e-4	1.00e-4	0.309	0.121	0.106	0.306	0.165
8q96[66]	0N4R tau	human, extracted	Cryo-EM	6.00e-4	8.30e-3	2.90e-3	1.28e-2	1.00e-4	0.156	0.102	0.112	0.119	0.173
8q92[66]	1N4R tau	human, extracted	Cryo-EM	1.00e-4	9.60e-3	3.40e-3	1.00e-4	1.00e-4	0.217	9.79e-2	0.108	0.350	0.168
6qjh[67]	2N4R tau	recombinant	Cryo-EM	4.00e-4	5.00e-4	2.00e-4	1.00e-4	0.103	0.197	0.181	0.195	0.273	0.288
6qjm[67]	2N4R tau	recombinant	Cryo-EM	1.00e-4	2.00e-4	1.00e-4	1.00e-4	1.00e-4	0.240	0.222	0.238	0.292	0.346
6qjp[67]	2N4R tau	recombinant	Cryo-EM	1.00e-4	1.00e-4	1.00e-4	1.00e-4	1.00e-4	0.252	0.235	0.251	0.276	0.363
6nwp[68]	2N4R tau	human, extracted	Cryo-EM	1.00e-4	2.50e-3	6.50e-3	1.00e-4	2.09e-2	0.231	0.135	0.115	0.234	0.099
8caq[61]	2N4R tau	human, extracted	Cryo-EM	1.00e-4	2.70e-3	7.00e-3	1.00e-4	2.25e-2	0.227	0.132	0.112	0.22967	0.097
8byn[69]	2N4R tau	human, extracted	Cryo-EM	1.00e-4	3.00e-4	9.00e-4	1.00e-4	2.25e-2	0.227	0.163	0.143	0.25199	0.097
6nwq[68]	2N4R tau	human, extracted	Cryo-EM	9.00e-4	2.00e-4	9.00e-4	3.00e-4	3.20e-3	0.191	0.211	0.181	0.19895	0.162

Table S6. Amyloid fibril/protofilament structures of 4R tau considering L ≥ 1 deposited in the PDB and listed in the *Amyloid Atlas*.[1]

PDB ID	Protein	Fibril origin	Method	*p* (one-tail Fisher's exact test)					*κ* (Cohen's kappa coefficient test)				
				ZYGGR.	AGGRES.	TANGO	WALTZ	PASTA2.0	ZYGGR.	AGGRES.	TANGO	WALTZ	PASTA2.0
8cax[61]	2N4R tau	human, extracted	Cryo-EM	1.00e-4	3.00e-3	8.20e-3	1.00e-4	2.60e-2	0.218	0.127	0.108	0.243	0.092
6tjo[62]	2N4R tau	human, extracted	Cryo-EM	1.00e-4	4.24e-2	1.98e-2	1.00e-4	4.00e-4	0.290	0.073	0.082	0.212	0.143
7p6d[63]	2N4R tau	human, extracted	Cryo-EM	1.00e-4	1.40e-3	1.33e-2	1.00e-4	4.00e-4	0.256	0.106	0.075	0.315	0.121
8orf[64]	1N4R tau	human, seeded	Cryo-EM	1.00e-4	2.10e-3	4.20e-3	1.00e-4	3.70e-3	0.296	0.117	0.102	0.259	0.112
8org[64]	1N4R tau	human, seeded	Cryo-EM	1.00e-4	1.00e-3	2.30e-3	1.00e-4	1.80e-3	0.259	0.135	0.118	0.293	0.129
6tjx[62]	2N4R tau	human, extracted	Cryo-EM	1.00e-4	7.35e-2	3.60e-2	1.00e-4	1.20e-3	0.307	0.057	0.067	0.284	0.119
7p6e[63]	2N4R tau	human, extracted	Cryo-EM	1.00e-4	1.60e-3	3.40e-3	1.00e-4	1.00e-4	0.350	0.123	0.108	0.310	0.168
7p65[63]	2N4R tau	human, extracted	Cryo-EM	1.00e-4	7.03e-2	0.124	1.00e-4	1.10e-3	0.249	5.86e-2	4.50e-2	0.306	0.121
7u0z[65]	2N4R tau	human, extracted	Cryo-EM	1.00e-4	0.703	0.124	1.00e-4	1.10e-3	0.249	5.86e-2	4.50e-2	0.324	0.121
7p66[63]	2N4R tau	human, extracted	Cryo-EM	1.00e-4	1.97e-2	7.30e-3	1.00e-4	2.00e-4	0.266	7.95e-2	8.90e-2	0.284	0.141
7p67[63]	2N4R tau	human, extracted	Cryo-EM	1.00e-4	1.54e-2	5.60e-3	1.00e-4	1.00e-4	0.280	8.57e-2	9.53e-2	0.318	0.150
7p68[63]	2N4R tau	human, extracted	Cryo-EM	1.00e-4<br>1.00e-4	1.64e-2<br>6.71e-2	6.00e-3<br>3.26e-2	1.00e-4<br>1.00e-4	1.00e-4<br>1.00e-4	0.259<br>0.273	8.41e-2<br>5.99e-2	9.36e-2<br>6.91e-2	0.314<br>0.273	0.148<br>0.145
7p6a[63]	2N4R tau	human, extracted	Cryo-EM	1.00e-4	5.30e-3	9.40e-3	1.00e-4	2.00e-4	0.252	9.51e-2	8.32e-2	0.321	0.133
7p6b[63]	2N4R tau	human, extracted	Cryo-EM	1.00e-4	4.60e-3	8.30e-3	1.00e-4	2.00e-4	0.262	9.83e-2	8.60e-2	0.330	0.137

7p6c[63]	2N4R tau	human, extracted	Cryo-EM	1.00e-4	5.00e-3	8.80e-3	1.00e-4	2.00e-4	0.259	9.67e-2	8.46e-2	0.308	0.135
8q96[66]	0N4R tau	human, extracted	Cryo-EM	1.00e-4	1.26e-2	4.50e-3	2.59e-2	1.00e-4	0.207	9.07e-2	0.100	0.102	0.157
8q92[66]	1N4R tau	human, extracted	Cryo-EM	1.00e-4	1.10e-2	3.90e-3	1.00e-4	1.00e-4	0.210	9.42e-2	0.104	0.360	0.162
6qjh[67]	2N4R tau	recombinant	Cryo-EM	9.00e-4	9.00e-4	3.00e-4	1.00e-4	1.00e-4	0.176	0.165	0.177	0.253	0.264
6qjm[67]	2N4R tau	recombinant	Cryo-EM	1.00e-4	2.00e-4	1.00e-4	1.00e-4	1.00e-4	0.240	0.222	0.238	0.292	0.346
6qjp[67]	2N4R tau	recombinant	Cryo-EM	1.00e-4	1.00e-4	1.00e-4	1.00e-4	1.00e-4	0.252	0.235	0.251	0.276	0.363
6nwp[68]	2N4R tau	human, extracted	Cryo-EM	1.00e-4	4.00e-3	9.60e-3	1.00e-4	2.98e-2	0.210	0.122	0.103	0.256	0.088
8caq[61]	2N4R tau	human, extracted	Cryo-EM	1.00e-4	4.30e-3	1.03e-2	1.00e-4	3.19e-2	0.207	0.119	0.101	0.252	0.086
8byn[69]	2N4R tau	human, extracted	Cryo-EM	1.00e-4	5.00e-4	1.30e-3	1.00e-4	2.98e-2	0.211	0.151	0.132	0.278	0.088
6nwq[68]	2N4R tau	human, extracted	Cryo-EM	4.00e-4	2.00e-3	5.50e-3	1.00e-4	1.79e-2	0.182	0.141	0.120	0.243	0.104

Supplementary References

1. Sawaya, M. R., Hughes, M. P., Rodriguez, J. A., Riek, R. & Eisenberg, D. S. The expanding amyloid family: Structure, stability, function, and pathogenesis. *Cell* **184**, 4857–4873 (2021).
2. Lührs, T. *et al.* 3D structure of Alzheimer's amyloid-β(1–42) fibrils. *Proceedings of the National Academy of Sciences* **102**, 17342–17347 (2005).
3. Qiang, W., Yau, W.-M., Luo, Y., Mattson, M. P. & Tycko, R. Antiparallel β-sheet architecture in Iowa-mutant β-amyloid fibrils. *Proceedings of the National Academy of Sciences* **109**, 4443–4448 (2012).
4. Paravastu, A. K., Leapman, R. D., Yau, W.-M. & Tycko, R. Molecular structural basis for polymorphism in Alzheimer's β-amyloid fibrils. *Proceedings of the National Academy of Sciences* **105**, 18349–18354 (2008).
5. Cerofolini, L. *et al.* Mixing Aβ(1–40) and Aβ(1–42) peptides generates unique amyloid fibrils. *Chemical Communications* **56**, 8830–8833 (2020).
6. Sgourakis, N. G., Yau, W.-M. & Qiang, W. Modeling an In-Register, Parallel "Iowa" Aβ Fibril Structure Using Solid-State NMR Data from Labeled Samples with Rosetta. *Structure* **23**, 216–227 (2015).
7. Lu, J.-X. *et al.* Molecular Structure of β-Amyloid Fibrils in Alzheimer's Disease Brain Tissue. *Cell* **154**, 1257–1268 (2013).
8. Schmidt, M. *et al.* Peptide dimer structure in an Aβ(1–42) fibril visualized with cryo-EM. *Proceedings of the National Academy of Sciences* **112**, 11858–11863 (2015).
9. Lee, M., Yau, W.-M., Louis, J. M. & Tycko, R. Structures of brain-derived 42-residue amyloid-β fibril polymorphs with unusual molecular conformations and intermolecular interactions. *Proceedings of the National Academy of Sciences* **120**, (2023).
10. Ghosh, U., Thurber, K. R., Yau, W.-M. & Tycko, R. Molecular structure of a prevalent amyloid-β fibril polymorph from Alzheimer's disease brain tissue. *Proceedings of the National Academy of Sciences* **118**, (2021).
11. Kollmer, M. *et al.* Cryo-EM structure and polymorphism of Aβ amyloid fibrils purified from Alzheimer's brain tissue. *Nat Commun* **10**, 4760 (2019).
12. Leistner, C. *et al.* The in-tissue molecular architecture of β-amyloid pathology in the mammalian brain. *Nat Commun* **14**, 2833 (2023).
13. Xiao, Y. *et al.* Aβ(1–42) fibril structure illuminates self-recognition and replication of amyloid in Alzheimer's disease. *Nat Struct Mol Biol* **22**, 499–505 (2015).
14. Colvin, M. T. *et al.* Atomic Resolution Structure of Monomorphic Aβ $_{42}$ Amyloid Fibrils. *J Am Chem Soc* **138**, 9663–9674 (2016).
15. Wälti, M. A. *et al.* Atomic-resolution structure of a disease-relevant Aβ(1–42) amyloid fibril. *Proceedings of the National Academy of Sciences* **113**, (2016).
16. Yang, Y. *et al.* Cryo-EM structures of amyloid-β 42 filaments from human brains. *Science (1979)* **375**, 167–172 (2022).
17. Stern, A. M. *et al.* Abundant Aβ fibrils in ultracentrifugal supernatants of aqueous extracts from Alzheimer's disease brains. *Neuron* **111**, 2012-2020.e4 (2023).
18. Yang, Y. *et al.* Cryo-EM structures of amyloid-β filaments with the Arctic mutation (E22G) from human and mouse brains. *Acta Neuropathol* **145**, 325–333 (2023).
19. Liu, D. *et al.* O-Glycosylation Induces Amyloid-β To Form New Fibril Polymorphs Vulnerable for Degradation. *J Am Chem Soc* **143**, 20216–20223 (2021).
20. Schütz, A. K. *et al.* Atomic-Resolution Three-Dimensional Structure of Amyloid β Fibrils Bearing the Osaka Mutation. *Angewandte Chemie International Edition* **54**, 331–335 (2015).
21. Gremer, L. *et al.* Fibril structure of amyloid-β(1–42) by cryo–electron microscopy. *Science (1979)* **358**, 116–119 (2017).

22. Tuttle, M. D. *et al.* Solid-state NMR structure of a pathogenic fibril of full-length human α-synuclein. *Nat Struct Mol Biol* **23**, 409–415 (2016).
23. Li, B. *et al.* Cryo-EM of full-length α-synuclein reveals fibril polymorphs with a common structural kernel. *Nat Commun* **9**, 3609 (2018).
24. Guerrero-Ferreira, R. *et al.* Cryo-EM structure of alpha-synuclein fibrils. *Elife* **7**, (2018).
25. Zhao, K. *et al.* Parkinson's disease-related phosphorylation at Tyr39 rearranges α-synuclein amyloid fibril structure revealed by cryo-EM. *Proceedings of the National Academy of Sciences* **117**, 20305–20315 (2020).
26. Zhao, K. *et al.* Parkinson's disease associated mutation E46K of α-synuclein triggers the formation of a distinct fibril structure. *Nat Commun* **11**, 2643 (2020).
27. Sun, Y. *et al.* Cryo-EM structure of full-length α-synuclein amyloid fibril with Parkinson's disease familial A53T mutation. *Cell Res* **30**, 360–362 (2020).
28. Ni, X., McGlinchey, R. P., Jiang, J. & Lee, J. C. Structural Insights into α-Synuclein Fibril Polymorphism: Effects of Parkinson's Disease-Related C-Terminal Truncations. *J Mol Biol* **431**, 3913–3919 (2019).
29. Boyer, D. R. *et al.* Structures of fibrils formed by α-synuclein hereditary disease mutant H50Q reveal new polymorphs. *Nat Struct Mol Biol* **26**, 1044–1052 (2019).
30. Guerrero-Ferreira, R. *et al.* Two new polymorphic structures of human full-length alpha-synuclein fibrils solved by cryo-electron microscopy. *Elife* **8**, (2019).
31. Boyer, D. R. *et al.* The α-synuclein hereditary mutation E46K unlocks a more stable, pathogenic fibril structure. *Proceedings of the National Academy of Sciences* **117**, 3592–3602 (2020).
32. Schweighauser, M. *et al.* Structures of α-synuclein filaments from multiple system atrophy. *Nature* **585**, 464–469 (2020).
33. Long, H. *et al.* Wild-type α-synuclein inherits the structure and exacerbated neuropathology of E46K mutant fibril strain by cross-seeding. *Proceedings of the National Academy of Sciences* **118**, (2021).
34. Sun, Y. *et al.* The hereditary mutation G51D unlocks a distinct fibril strain transmissible to wild-type α-synuclein. *Nat Commun* **12**, 6252 (2021).
35. Hojjatian, A. *et al.* Tau induces formation of α-synuclein filaments with distinct molecular conformations. *Biochem Biophys Res Commun* **554**, 145–150 (2021).
36. Lövestam, S. *et al.* Seeded assembly *in vitro* does not replicate the structures of α-synuclein filaments from multiple system atrophy. *FEBS Open Bio* **11**, 999–1013 (2021).
37. Frieg, B. *et al.* Quaternary structure of patient-homogenate amplified α-synuclein fibrils modulates seeding of endogenous α-synuclein. *Commun Biol* **5**, 1040 (2022).
38. Sun, C. *et al.* Cryo-EM structure of amyloid fibril formed by α-synuclein hereditary A53E mutation reveals a distinct protofilament interface. *Journal of Biological Chemistry* **299**, 104566 (2023).
39. Fan, Y. *et al.* Conformational change of α-synuclein fibrils in cerebrospinal fluid from different clinical phases of Parkinson's disease. *Structure* **31**, 78-87.e5 (2023).
40. Tao, Y. Q., Sun, Y. P., Liu, C. & Li, D. Heparin-induced alpha-synuclein fibrils polymorph 1. Preprint at (2022).
41. Tao, Y. Q. *et al.* Heparin-remodelled alpha-synuclein fibrils. Preprint at (2022).
42. Tao, Y. Q., Zhao, Q. Y., Liu, C. & Li, D. alpha-synuclein fibril-F0502B complex. Preprint at https://doi.org/10.1038/nsb1203-980 (2023).
43. Wu, K.-P. & Huang, J. Y.-C. CryoEM structure of human alpha-synuclein A53T fibril. *wwPDB* Preprint at (2023).
44. Huang, J. Y.-C. & Wu, K.-P. CryoEM structure of human alpha-synuclein A53T fibril induced by calcium ions. Preprint at (2023).
45. Zhao, Q. *et al.* Structural Insights of Fe3+ Induced α-synuclein Fibrillation in Parkinson's Disease. *J Mol Biol* **435**, 167680 (2023).

46. Zhang, S. *et al.* Conformational Dynamics of an α-Synuclein Fibril upon Receptor Binding Revealed by Insensitive Nuclei Enhanced by Polarization Transfer-Based Solid-State Nuclear Magnetic Resonance and Cryo-Electron Microscopy. *J Am Chem Soc* **145**, 4473–4484 (2023).
47. Tao, Y. Q., Zhao, Q. Y., Liu, C. & Li, D. CR-bound alpha-synuclein fibrils. Preprint at (2023).
48. Tao, Y. Q., Zhao, Q. Y., Liu, C. & Li, D. EB-bound alpha-synuclein fibrils. Preprint at (2023).
49. Tao, Y. Q., Zhao, Q. Y., Liu, C. & Li, D. ThT-bound alpha-synuclein fibrils conformation 1. Preprint at (2023).
50. Tao, Y. Q., Zhao, Q. Y., Liu, C. & Li, D. ThT-bound alpha-synuclein fibrils conformation 2. Preprint at https://doi.org/https://doi.org/10.2210/pdb7YNN/pdb (2023).
51. Tao, Y. Q., Zhao, Q. Y., Liu, C. & Li, D. PiB-bound alpha-synuclein fibrils conformation 1. Preprint at https://doi.org/https://doi.org/10.2210/pdb7YNO/pdb (2023).
52. Tao, Y. Q., Zhao, Q. Y., Liu, C. & Li, D. BF227-bound alpha-synuclein fibrils. Preprint at https://doi.org/https://doi.org/10.2210/pdb7YNP/pdb (2023).
53. Tao, Y. Q., Zhao, Q. Y., Liu, C. & Li, D. PiB-bound alpha-synuclein fibrils conformation 2. Preprint at https://doi.org/https://doi.org/10.2210/pdb7YNQ/pdb (2023).
54. Tao, Y. Q., Zhao, Q. Y., Liu, C. & Li, D. C05-03-bound alpha-synuclein fibrils. Preprint at https://doi.org/https://doi.org/10.2210/pdb7YNR/pdb (2023).
55. Tao, Y. Q., Zhao, Q. Y., Liu, C. & Li, D. SIL5-bound alpha-synuclein fibrils. Preprint at https://doi.org/https://doi.org/10.2210/pdb7YNS/pdb (2023).
56. Tao, Y. Q., Zhao, Q. Y., Liu, C. & Li, D. pFTAA-bound alpha-synuclein fibrils. Preprint at https://doi.org/https://doi.org/10.2210/pdb7YNT/pdb (2023).
57. Frieg, B. *et al.* The 3D structure of lipidic fibrils of α-synuclein. *Nat Commun* **13**, 6810 (2022).
58. Yang, Y. *et al.* Structures of α-synuclein filaments from human brains with Lewy pathology. *Nature* **610**, 791–795 (2022).
59. Sokratian Arpine *et al.* Structural and functional landscape of α-synuclein fibril conformations amplified from cerebrospinal fluid. Preprint at https://doi.org/https://doi.org/10.1101/2022.07.13.499896 (2023).
60. Dhavale, D. D. *et al.* Structure of alpha-synuclein fibrils derived from human Lewy body dementia tissue. *Nat Commun* **15**, 2750 (2024).
61. Qi, C. *et al.* Identical tau filaments in subacute sclerosing panencephalitis and chronic traumatic encephalopathy. *Acta Neuropathol Commun* **11**, 74 (2023).
62. Zhang, W. *et al.* Novel tau filament fold in corticobasal degeneration. *Nature* **580**, 283–287 (2020).
63. Shi, Y. *et al.* Structure-based classification of tauopathies. *Nature* **598**, 359–363 (2021).
64. Tarutani, A. *et al.* Cryo-EM structures of tau filaments from SH-SY5Y cells seeded with brain extracts from cases of Alzheimer's disease and corticobasal degeneration. *FEBS Open Bio* **13**, 1394–1404 (2023).
65. Chang, A. *et al.* Homotypic fibrillization of TMEM106B across diverse neurodegenerative diseases. *Cell* **185**, 1346-1355.e15 (2022).
66. Schweighauser, M. *et al.* Cryo-EM structures of tau filaments from the brains of mice transgenic for human mutant P301S Tau. *Acta Neuropathol Commun* **11**, 160 (2023).
67. Zhang, W. *et al.* Heparin-induced tau filaments are polymorphic and differ from those in Alzheimer's and Pick's diseases. *Elife* **8**, (2019).
68. Falcon, B. *et al.* Novel tau filament fold in chronic traumatic encephalopathy encloses hydrophobic molecules. *Nature* **568**, 420–423 (2019).

69. Shi, Y., Ghetti, B., Goedert, M. & Scheres, S. H. W. Cryo-EM Structures of Chronic Traumatic Encephalopathy Tau Filaments with PET Ligand Flortaucipir. *J Mol Biol* **435**, 168025 (2023).
